# Cost-effectiveness of combined veno-arterial extracorporeal membrane oxygenation and Impella support (ECPELLA) in infarct-related cardiogenic shock

**DOI:** 10.1093/ehjopen/oeag064

**Published:** 2026-04-15

**Authors:** Priyanka Boettger, Andrija Matetic, Jamschid Sedighi, Bernhard Unsoeld, Christoph Struenck, Michael Buerke, Dirk Westermann, Samuel Sossalla

**Affiliations:** Department of Internal Medicine I, Cardiology, Angiology and Intensive Care Medicine, Justus-Liebig-University, Klinikstrasse 33, 35392 Giessen, Hessen, Germany; Department of Health Policy, London School of Economics and Political Science, London, UK; Cardio-Pulmonary Institute (CPI), 35392 Giessen, Hessen, Germany; Department of Health Policy, London School of Economics and Political Science, London, UK; Department of Cardiology, University Hospital Split, 21000 Split, Croatia; Department of Internal Medicine I, Cardiology, Angiology and Intensive Care Medicine, Justus-Liebig-University, Klinikstrasse 33, 35392 Giessen, Hessen, Germany; Cardio-Pulmonary Institute (CPI), 35392 Giessen, Hessen, Germany; Department of Internal Medicine I, Cardiology, Angiology and Intensive Care Medicine, Justus-Liebig-University, Klinikstrasse 33, 35392 Giessen, Hessen, Germany; Department of Social Sciences, Faculty of Humanities, University of Siegen, 57076 Siegen, North Rhine-Westphalia, Germany; Department of Internal Medicine II, Heart-Center-Südwestfalen, Cardiology, Angiology and Intensive Care Medicine, St.Marien-Krankenhaus, 57072 Siegen, North Rhine-Westphalia, Germany; Department of Cardiology and Angiology, University Heart Center Freiburg, Faculty of Medicine, University of Freiburg, 79106 Freiburg, Baden-Württemberg, Germany; Department of Internal Medicine I, Cardiology, Angiology and Intensive Care Medicine, Justus-Liebig-University, Klinikstrasse 33, 35392 Giessen, Hessen, Germany; Cardio-Pulmonary Institute (CPI), 35392 Giessen, Hessen, Germany; Department of Cardiology, Kerckhoff Clinic, Campus Kerckhoff of the Justus-Liebig-University Giessen, 61231 Bad Nauheim, Hessen, Germany

**Keywords:** Cardiogenic shock, ECPELLA, Impella, VA-ECMO, Cost-effectiveness, QALY

## Abstract

**Aims:**

Cardiogenic shock carries high early mortality. Combined veno-arterial extracorporeal membrane oxygenation and Impella support (ECPELLA) may improve outcomes but is costly and unevenly available across Europe. Its incremental clinical and economic value compared with guideline-directed medical therapy (GDMT) remains uncertain.

**Methods:**

We developed a calibrated partitioned-survival model comparing ECPELLA with GDMT over a 10-year horizon from a statutory health insurance payer perspective (2024€ reference costing environment). Costs, utilities, and survival inputs were derived from contemporary randomized trials and multinational registries. Uncertainty was assessed using probabilistic, deterministic, scenario, and value-of-information analyses. European cost-environment scenarios were explored to assess transferability

**Results:**

ECPELLA yielded 4.05 quality-adjusted life-years (QALYs) at €195 000 vs. 3.10 QALYs at €95 000 for GDMT (incremental cost €100 000; incremental QALY 0.95), resulting in an incremental cost-effectiveness ratio (ICER) of €105 263 per QALY. Device and intensive care unit (ICU) costs and early survival effects were the main drivers of uncertainty. Cost-effectiveness improved in patients younger than 60 years, those with reversible myocardial dysfunction, early cannulation, and treatment in high-volume centres (ICER €60 000–80 000 per QALY), whereas neutral survival effects produced ICERs exceeding €300 000 per QALY. Value-of-information analysis indicated residual decision uncertainty.

**Conclusion:**

ECPELLA increased quality-adjusted survival at higher cost. In the base-case analysis, the ICER was €105 263 per QALY gained. In predefined scenario analyses, ICERs were lower in younger patients, those with recovery potential, early cannulation, and high-volume centres. Residual decision uncertainty was observed in value-of-information analyses.

## Graphical Abstract

**Graphical Abstract:**
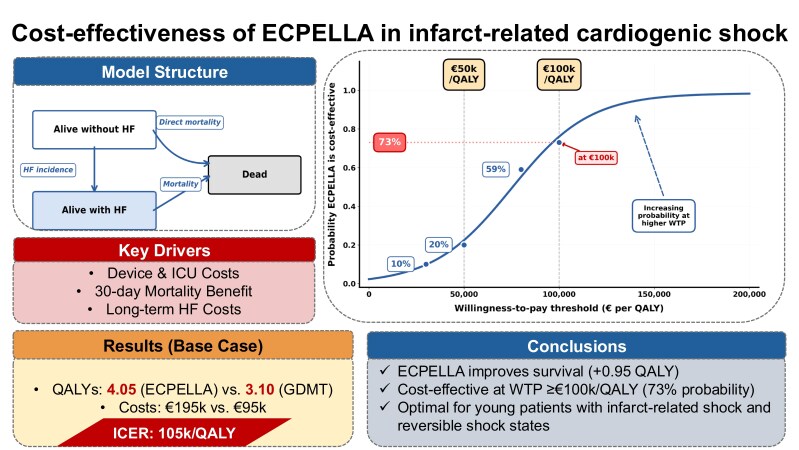

Key learning points *What is already known*

- Cardiogenic shock carries extremely high early mortality, and conventional GDMT alone yields poor outcomes despite contemporary PCI and ICU management.
- VA-ECMO improves haemodynamics but may worsen left-ventricular loading, while adjunctive Impella (‘ECPELLA’) has been associated with lower short-term mortality in observational cohorts and the DanGer–Shock trial.
- ECPELLA is highly resource-intensive, with device and ICU costs frequently exceeding €100 000 per patient, and its overall cost-effectiveness relative to GDMT has remained uncertain.

*What this study adds*

- This analysis provides the first structured cost-utility evaluation of ECPELLA vs. GDMT using a calibrated partitioned-survival model with probabilistic and value-of-information methods.
- ECPELLA improves quality-adjusted survival (ΔQALY ≈ 0.95) but at substantially higher cost (ΔCost ≈ €100 000), yielding an ICER of €105 263/QALY, which exceeds commonly referenced willingness-to-pay benchmarks in unselected populations.
- Cost-effectiveness estimates were lower in predefined scenario analyses involving younger age, reversible aetiologies, early cannulation, and high-volume centres. Expected value of partial perfect information was highest for survival effect and device cost parameters.

## Introduction

Cardiogenic shock (CS) is characterized by circulatory collapse due to acute cardiac dysfunction, often complicating myocardial infarction.^1,2^ Despite advances in intensive care, 30-day mortality remains approximately 50% across contemporary high-income health systems.^3^ Current ESC and AHA Guidelines recommend revascularization in combination with inotropic and vasopressor support.^2,4^ Further, rapid escalation to mechanical circulatory support (MCS) may be required when end-organ perfusion cannot be maintained by pharmacologic support alone.^2^ The best support modality is debated. Randomized trials (ECLS-SHOCK,^1^ ECMO-CS^5^) found no early survival benefit from routine VA-ECMO in AMI-associated CS.^1,2^ However, retrospective data suggest that unloading the left ventricle improves outcomes: Combining Impella with ECMO (‘ECPELLA’) is associated with lower 30-day mortality (HR 0.79) than ECMO alone.^6,7^ Moreover, the DanGer–Shock trial reported that adding Impella CP to standard care reduced 180-day mortality (45.8% vs. 58.5%, HR 0.74) at the expense of more device-related complications.^8,9^ In practice, ECPELLA is increasingly used in therapy-refractory CS, especially in specialized centres.^10,11^ However, access to advanced temporary MCS varies substantially across European health systems, reflecting differences in institutional capacity, centre volume, and reimbursement structures. As a result, adoption is concentrated in high-volume tertiary centres, and real-world implementation remains heterogeneous.^12^ Alongside clinical outcomes, understanding the economic implications of ECPELLA is critical.^13^ Mechanical circulatory support devices and prolonged ICU care are highly resource-intensive. Published reviews report mean total hospital cost per ECMO patient >€100k.^14^ Impella devices themselves have large upfront costs (tens of thousands of Euros) and also require specialized care. Prior cost-effectiveness studies of Impella vs. ECMO suggest comparable or lower costs for Impella in some settings.^15^ However, no comprehensive model has evaluated ECMO + Impella vs. medical therapy in CS. Despite the growing adoption of percutaneous MCS in CS, the long-term clinical and economic value of ECPELLA compared with GDMT remains uncertain.^6,16^ Contemporary evidence from randomized trials and registries suggests improved short-term survival with mechanical support, yet robust data on downstream morbidity, quality of life, and lifetime healthcare costs are lacking.^17,18^

Addressing this evidence gap is essential for clinicians and policymakers, particularly because ECPELLA use is largely concentrated in high-volume hospitals and university centres, while access, organizational capacity, and shock-team expertise vary widely across European health systems. This uneven adoption means that GDMT continues to serve as the real-world comparator in most care settings, highlighting the need to quantify the incremental value of ECPELLA within contemporary clinical pathways.^19^

To inform equitable and sustainable decision-making, we therefore evaluated the clinical and economic implications of ECPELLA compared with real-world GDMT in infarct-related CS. Our modelling approach integrates short-term survival, downstream heart failure (HF) burden, device-related morbidity, and long-term costs to determine whether the potential physiological benefits of ECPELLA translate into meaningful value within contemporary high-resource health systems characterized by heterogeneous access to advanced MCS.

## Methods

### Model overview

**Figure 1:**
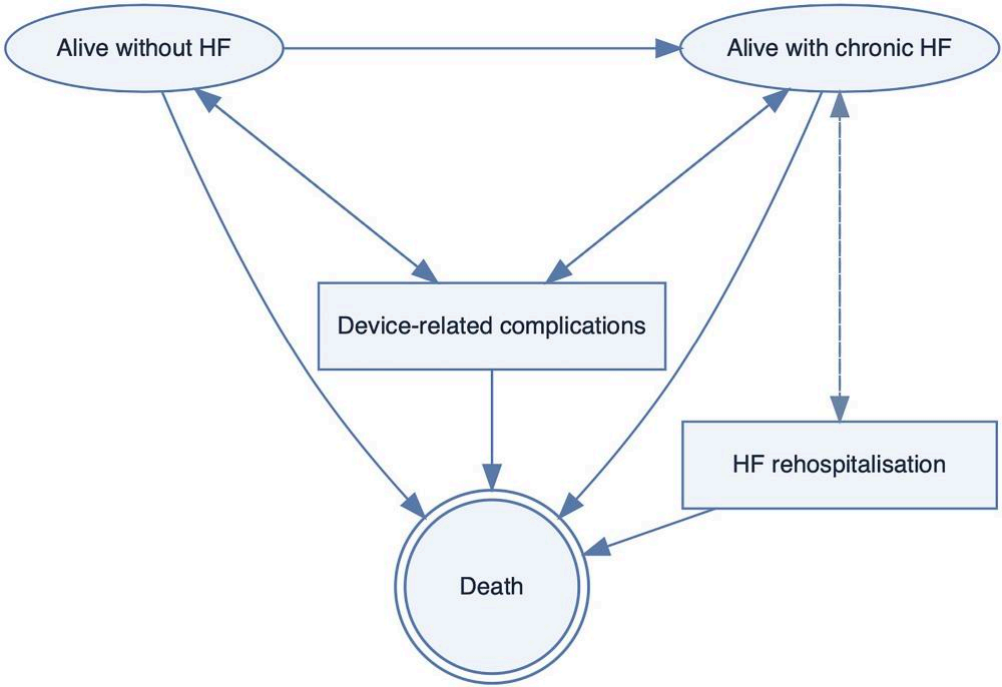
**Partitioned survival model structure for ECPELLA vs. GDMT**. Patients enter the model alive without heart failure (HF) following the index hospitalization and transition between three health states: alive without HF, alive with HF, and dead. Overall survival and HF-free survival were derived from calibrated parametric survival models incorporating a proportional hazards treatment effect for ECPELLA. Monthly cycles were applied during the first year and annual cycles thereafter over a 10-year analytic horizon. Costs and outcomes were evaluated from the German statutory health insurance perspective and discounted at 3% annually. The model captures acute device-related morbidity and long-term HF progression.

We developed a partitioned-survival model (PSM) to compare ECPELLA (VA-ECMO + Impella unloading) with guideline-directed medical therapy (GDMT) in infarct-related CS.^20^ Overall survival and non-HF survival were modelled directly, and time spent in the health states ‘alive without HF’, ‘alive with HF’, and ‘dead’ was derived from the area under and between survival curves^21^ (*Figure 1*, Supplementary material online, *Figure S1*). Monthly cycles were used in year 1 (including an acute 0–6-month tunnel phase to capture early mortality and temporary utility decrements), followed by annual cycles.^22^

A 10-year analytic horizon and a 3% annual discount rate were applied to costs and health outcomes, in line with IQWiG and European guidance.^23^ For clarity, the modelling workflow of our partitioned-survival framework is summarized in *Figure 1*. Baseline survival under GDMT was reconstructed from contemporary randomized trials and registry data and extrapolated using calibrated parametric survival models within a macro-based implementation of the partitioned-survival framework. The treatment effect associated with ECPELLA was then applied under a proportional hazards assumption to generate treatment-specific survival trajectories, from which health-state occupancy and accumulated costs and quality-adjusted life-years were derived. Full methodological details, including survival modelling, cost derivation, uncertainty analyses, and validation procedures, are provided in the Supplementary Methods. Model development and scenario definitions were prespecified in a health economic analysis plan.

#### Perspective, setting, and population

The analysis was conducted from the perspective of the German statutory health insurance (GKV), with all costs expressed in 2024 Euros (price year 2024). Germany was used as the reference reimbursement setting because detailed DRG-based hospital costing data and institutional micro-costing inputs were available. Unit cost parameters were benchmarked against published Western European ICU and ECMO cost analyses, which demonstrate substantial cross-country variability but broadly overlapping cost ranges. Sensitivity and scenario analyses were performed to explore the impact of variation in acute care resource use and pricing on cost-effectiveness outcomes. The simulated cohort represents adults (mean age approximately 65–70 years) with infarct-related CS who underwent early revascularization and received intensive care management in tertiary hospitals.

#### Comparator definition (guideline-directed medical therapy, GDMT)

Guideline-directed medical therapy was defined as contemporary intensive medical management for infarct-related CS without MCS. It comprised early revascularization, vasoactive and inotropic therapy, ventilatory support, renal replacement therapy when indicated, and ICU-based monitoring in accordance with current guideline recommendations.^2,4,24^ Temporary MCS, including intra-aortic balloon pump, veno-arterial extracorporeal membrane oxygenation, and microaxial flow pumps, was excluded from the GDMT comparator. Device-based circulatory support was evaluated exclusively in the ECPELLA arm. Baseline GDMT survival was calibrated to contemporary randomized trials and registries reflecting modern revascularization and ICU practice.^5,11,17^

### Clinical effectiveness

Baseline overall survival under GDMT was derived from contemporary randomized control arms^1,5^ and validated against registry cohorts to reflect modern revascularization and intensive care practice. Kaplan–Meier survival curves were digitized and fitted using parametric survival models, with model selection guided by statistical fit criteria and clinical plausibility. External calibration ensured alignment with observed 30-day and 1-year mortality rates in contemporary PCI-treated CS populations.^1,5^

In the absence of a direct randomized ECPELLA vs. GDMT comparison, the relative mortality reduction observed in the DanGer–Shock trial^9^ was applied to the calibrated GDMT survival curve under a proportional hazard assumption. The key clinical input parameters and uncertainty ranges used in the base-case and sensitivity analyses are summarized in *Table 1*. Observational ECPELLA estimates were not used to parameterize the base-case treatment effect but were explored in scenario analyses. A detailed description of the hierarchical derivation of survival inputs, calibration procedures, and implementation of treatment effects is provided in the Supplementary Methods. Incident HF was derived from longitudinal registry data,^26^ and utilities for post-ACS and HF states were informed by EQ-5D-based survivorship data,^27^ with a temporary short-term disutility applied during the acute recovery phase (see Supplementary material online, *Table S3*).

**Table 1:** Model input parameters for base-case and sensitivity analyses

Parameter	Base value	Range (OWSA)	Distribution (PSA)	Source/notes
*Clinical parameters*				
**30-day mortality (GDMT)**	0.50	0.40–0.60	Beta	Calibrated to ECLS–SHOCK registry data^1^
**ECPELLA vs. GDMT-hazard ratio (mortality)**	0.74 (95% CI 0.55–0.99)	0.55–0.99	Log-normal	Møller et al., 2024 (DanGer–Shock trial)^9^
**Annual post-discharge mortality (GDMT)**	0.15	0.10–0.20	Beta	Contemporary CS registry extrapolation^25^
**Annual HF incidence (post-CS)**	0.08	0.05–0.12	Beta	Lauridsen et al., 2021^26^
*Utilities (EQ-5D based)*				
**Utility, no HF (post-MI recovery)**	0.80	0.75–0.85	Beta	Hall et al., 2025; Di Tanna et al. 2021^27,28^
**Utility, with HF (NYHA II–III)**	0.65	0.60–0.70	Beta	Hall et al., 2025; Di Tanna et al. 2021^27,28^
**Temporary utility decrement (acute tunnel, 0–6 months)**	−0.10	−0.05 to −0.15	Beta (bounded)	Post-ICU morbidity (Post-ICU Syndrome)
*Costs (2024€, payer perspective)*				
**Impella device + technical cost**	€57 770	€45 000–€65 000	Gamma	Rognoni et al., 2025^15^
**VA-ECMO device + setup cost (ICU days costed separately)**	€60 000	€50 000–€75 000	Gamma	Institutional micro-costing; Mishra et al., 2010^29,30^
**ICU day (including personnel)**	€2 000	€1 500–€3 000	Gamma	European ICU cost studies^31,32^
**Annual HF management (after year 1)**	€3 000	€1 000–€5 000	Gamma	European HF cost studies^29,33,34^
**HF rehospitalization (per event)**	€5 000	€3 000–€7 000	Gamma	European HF registries, payer estimates^29,34,35^
*Model parameters*				
**Discount rate (costs/effects)**	3% per year	0–6%	Fixed	International guidelines for economic evaluation^36^
**Time horizon**	10 years	—	Fixed	Reflects long-term survival convergence
**Perspective**	Payer	—	—	European Health system

### Cost inputs

Direct medical costs included device acquisition, ICU and ward length of stay, revascularization procedures, management of complications, and long-term HF care. Resource quantities were informed by contemporary European clinical trials and registry data. Unit costs were derived from the German statutory health insurance reference setting using DRG-based and institutional hospital accounting data. To contextualize these values within high-income European healthcare systems, unit costs were compared against published Western European ICU cost–unit accounting and ECMO episode–cost studies.^13,29^ Reported ICU per-diem costs across Western Europe range approximately between €1500 and €3500 per day, placing the base-case ICU per-diem (€2000) within the mid-range of published estimates. Device and ECMO episode costs similarly fall within ranges reported in European economic evaluations and micro-costing analyses (see Supplementary material online, *Table S2*).^31^ Intensive care unit costs were modelled using a bundled per-diem approach incorporating staffing, organ support (including mechanical ventilation and renal replacement therapy), vasoactive and inotropic therapy, diagnostics, and consumables. Acute ICU length of stay was modelled explicitly for both GDMT and ECPELLA arms (*Table 1*). To reflect the higher resource intensity associated with ECPELLA, including near-universal invasive ventilation, increased monitoring, and higher rates of renal replacement therapy, ICU per-diem costs in the ECPELLA arm were adjusted using an intensity multiplier applied to the GDMT per-diem.^10,18^ VA-ECMO costs were modelled as device and setup expenditures, with ICU days costed separately to avoid double counting. All costs were standardized to 2024€ using national health sector inflation indices applicable to the reference costing environment. Parameter ranges used in deterministic and probabilistic sensitivity analyses were selected to encompass variability observed in Western European cost studies. Detailed model parameters, uncertainty distributions, and the ICU per-diem micro-costing decomposition are provided in Supplementary material online, *Tables S1* and *S2*.

### Uncertainty and value-of-information analyses

Parameter uncertainty was explored using probabilistic sensitivity analysis (10 000 Monte Carlo simulations) and one-way sensitivity analyses for key parameters. In addition, a value-of-information (VOI) framework was applied to quantify the expected monetary consequences of decision uncertainty within the model. VOI methods estimate how much a decision-maker would gain, in monetary terms, if uncertainty in model parameters were reduced through additional evidence generation. In practical terms, value-of-information analysis estimates how much could be gained from reducing uncertainty in key model parameters and therefore indicates whether further clinical research is likely to be worthwhile before making funding or policy decisions. The expected value of perfect information (EVPI) represents the maximum potential benefit of eliminating all parameter uncertainty, reflecting the value of completely resolving the remaining uncertainty through additional research. The expected value of partial perfect information (EVPPI) quantifies the value of reducing uncertainty in specific parameter groups and therefore helps identify which parameters would benefit most from further evidence generation. Full specifications of parameter distributions, correlation structures, and VOI methods are provided in the Supplementary Methods. Because ICU resource use in CS is highly heterogeneous, we explored low-intensity ICU care scenarios with reduced per-diem costs and shorter ICU stays to reflect rapid stabilization and step-down to the ward.^37,38^

### Model validation

Model validation followed ISPOR–SMDM recommendations and included face, internal, external, and structural validation. A simplified Markov model served as an additional structural check. Detailed validation procedures and calibration targets are reported in the Supplementary material. The economic evaluation is reported in accordance with the Consolidated Health Economic Evaluation Reporting Standards (CHEERS 2022). A completed CHEERS checklist is provided in Supplementary material online, *Table S7*.^37,38^

### Transferability considerations

The base-case analysis adopts the perspective of the German statutory health insurance system because detailed DRG-based and institutional micro-costing data were available for acute episode costs. Clinical effectiveness inputs, including baseline survival, relative treatment effects, and HF progression, were derived from contemporary multinational trials and European registries, thereby reflecting outcomes across diverse high-income health systems. Unit cost parameters were benchmarked against published Western European ICU cost-unit accounting and ECMO micro-costing studies (see Supplementary material online, *Tables S1* and *S2*). Deterministic ranges were selected to encompass reported cross-country variability in device pricing, staffing costs, and acute-care resource use. Because unit costs are inherently jurisdiction-specific, cross-system cost variation was treated as an uncertainty domain rather than assuming uniform pricing across Europe. To further examine cross-country transferability, we conducted a predefined deterministic European cost-environment scenario analysis. Acute-care cost valuation (ICU and ward resource intensity) was varied by ±20%, and device procurement costs by ±15%, reflecting plausible cross-country variation reported in Western European cost-accounting and procurement studies. Incremental health outcomes (ΔQALY) were held constant to isolate the impact of cost-structure variation on incremental cost-effectiveness results.

The model structure, including the partitioned-survival framework, health-state definitions, and treatment-effect implementation, is independent of reimbursement system characteristics. Sensitivity and scenario analyses therefore allow interpretation of relative cost-effectiveness gradients across comparable high-resource health systems while maintaining transparency regarding the reference costing environment.

## Results 

Results are structured by base-case findings, followed by probabilistic and deterministic uncertainty analyses, scenario exploration, and value-of-information analysis.

### Base-case analysis

ECPELLA generated 4.05 QALYs at a total cost of €195 000 compared with 3.10 QALYs at €95 000 for GDMT, resulting in an incremental cost of €100 000 and an incremental gain of 0.95 QALYs. The corresponding incremental cost-effectiveness ratio (ICER) was €105 263 per QALY gained. Incremental net monetary benefit (INMB) values remained negative across commonly cited benchmark willingness-to-pay (WTP) thresholds and approached economic neutrality only at approximately €100 000 per QALY (see Supplementary material online, *Figure S2*). Base-case and scenario-specific outcomes are summarized in *Table 2*.

**Table 2:** Base-case and scenario cost-effectiveness results

*Scenario*	*ΔCost (€)*	*ΔQALY*	*ICER (€/QALY)*	*INMB @ €30 000 (€)*	*INMB @ €50 000 (€)*	*INMB @ €100 000 (€)*
*Base case (60-year-old STEMI-CS)*	100 000	0.95	105 263	−71 500	−52 500	−5 000
*Favourable (age 50, reversible cause, high-volume centre)*	105 000	1.60	65 625	−57 000	−25 000	55 000
*Unfavourable (no short-term benefit, HR = 1.0)*	100 000	0.30	333 333	−91 000	−85 000	−70 000

*Base case*: 60-year old STEMI-CS. *Favourable scenario*: age 50, reversible cause (e.g. myocarditis), high-volume centre. *Unfavourable*: no short-term benefit (ECPELLA HR = 1).

Notes: Costs are expressed in 2024€ from the German statutory health insurance (GKV) perspective. Results are discounted at 3% annually for both costs and outcomes. QALY, quality-adjusted life-year (utilities derived from EQ-5D-based inputs^1,9,27^); ICER, incremental cost-effectiveness ratio; INMB, incremental net monetary benefit; ECPELLA, veno-arterial extracorporeal membrane oxygenation plus Impella support; GDMT, guideline-directed medical therapy.

## Characterization of uncertainty

**Figure 2:**
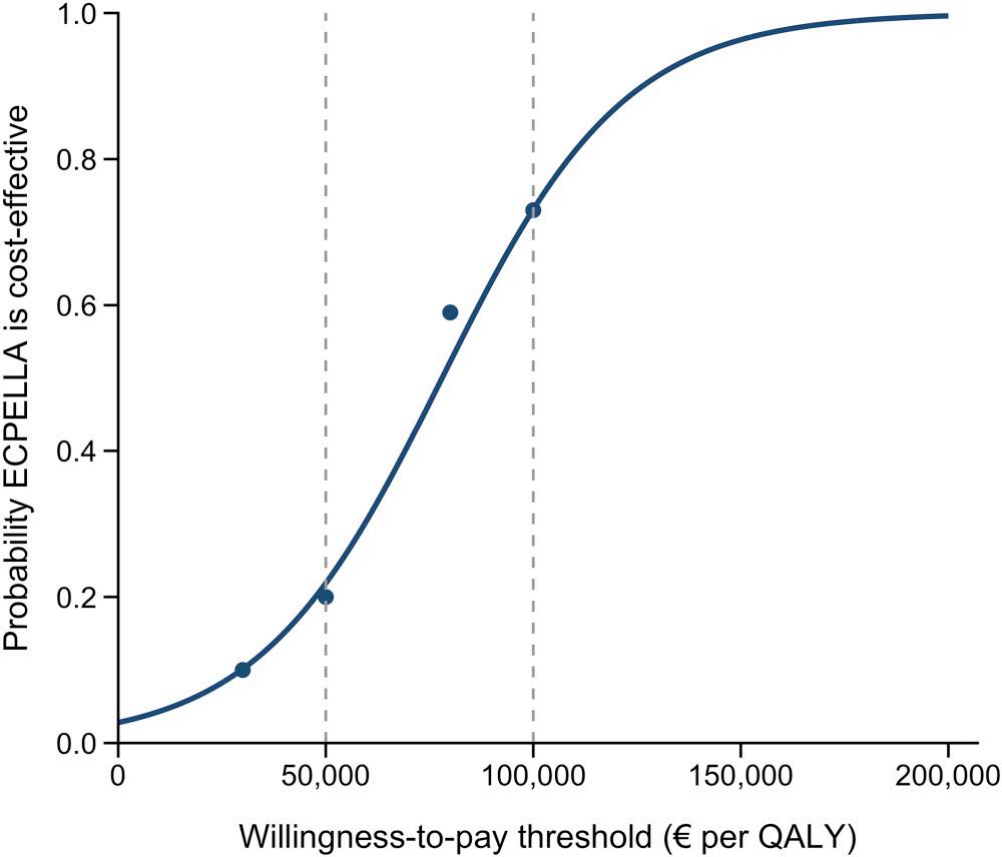
**Cost-effectiveness acceptability curve (CEAC) for ECPELLA vs. medical therapy.** The CEAC displays the probability that ECPELLA is cost-effective compared with guideline-directed medical therapy (GDMT) across a range of willingness-to-pay (WTP) thresholds per quality-adjusted life-year (QALY). The curve is based on the 10-year base-case analysis assuming a proportional hazards treatment effect for ECPELLA and 3% annual discounting of costs and outcomes. The probability of cost-effectiveness increases between €50 000 and €100 000 per QALY, reaching approximately 0.7–0.8 at €100 000 per QALY and approaching 1.0 at higher WTP values. Dashed vertical lines indicate selected benchmark WTP levels (€50 000 and €100 000 per QALY). Results are derived from 10 000 probabilistic simulations and illustrate the degree of decision uncertainty.

Because ICER confidence intervals are unstable when ΔQALY is small, uncertainty was assessed using probabilistic and net–benefit approaches. Cost-effectiveness acceptability curves (CEACs) showed that the probability of ECPELLA being cost-effective was ∼0.20 at €50 000/QALY, increasing to ∼0.73 at €100 000/QALY (*Figure 2*).^39^

## Clinical interpretation of base-case results

The incremental gain of 0.95 QALYs corresponds to roughly 11 quality-adjusted months. Modelled 30-day survival was 58% with ECPELLA vs. 48% with GDMT, and 1-year survival 46% vs. 38%. This absolute 8-percentage-point difference corresponds to an NNT of 13. These gains are clinically meaningful in a population with >50% early mortality and support targeted use in patients most likely to benefit.

## Secondary results

Beyond cost-effectiveness, ECPELLA improved clinical outcomes. The mean undiscounted survival gain was about 1 life-year (0.95 QALYs, ≈11 quality-adjusted months). Predicted survival was 58% vs. 48% at 30 days and 46% vs. 38% at 1 year, an 8-percentage-point absolute difference (number needed to treat 13). Incremental costs were driven mainly by device and ICU use (≈80% of ΔCost), whereas HF rehospitalization costs were modestly lower with ECPELLA. These benefits occurred at substantial additional cost, with conclusions robust across sensitivity and value-of-information analyses.

### Probabilistic sensitivity analysis

**Figure 3:**
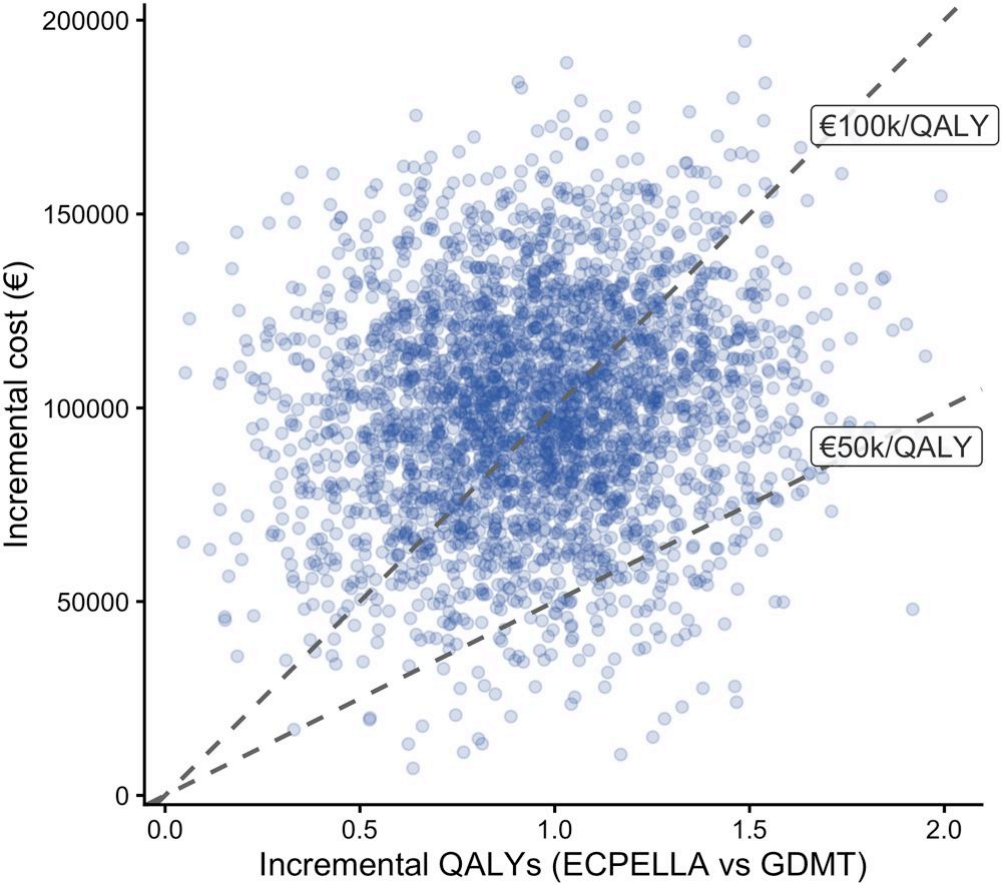
**Cost-effectiveness plane (probabilistic sensitivity analysis).** Each point represents one of 10 000 Monte Carlo simulations from the 10-year base-case analysis, assuming a proportional hazards treatment effect for ECPELLA and 3% annual discounting of costs and outcomes. Points in the northeast quadrant indicate that ECPELLA is more effective but also more costly than guideline-directed medical therapy (GDMT). The dashed lines denote willingness-to-pay (WTP) thresholds of €50 000 and €100 000 per quality-adjusted life-year (QALY). The clustering of simulations around the base-case incremental cost-effectiveness ratio (ICER €105 263/QALY) reflects the central estimate, whereas sparse upper-right outliers represent low-probability high-cost realizations..

Across 10 000 Monte Carlo simulations, all iterations fell in the northeast quadrant of the cost-effectiveness plane, indicating that ECPELLA was consistently more effective but more costly than GDMT (*Figure 3*). Most simulations clustered tightly around the base-case ICER, with a small number of high-cost, low-effectiveness outliers. The CEAC showed that the probability of ECPELLA being cost-effective was 20% at €50 000/QALY, increasing to 59% at €80 000/QALY and 73% at €100 000/QALY (*Table 3*). These findings indicate substantial decision uncertainty at commonly cited benchmark willingness-to-pay thresholds, with increasing probability of cost-effectiveness at higher WTP values.^40,41^

**Table 3:** Probabilistic sensitivity analysis summary (10 000 iterations)

*WTP threshold (€/QALY)*	*Probability cost-effective P(CE)*	*Median ICER (€/QALY)*	*Decision-error probability 1-P(CE)*
*30 000€*	0.10	105 000	0.90
*50 000€*	0.20	104 000	0.80
*80 000€*	0.59	103 000	0.41
*100 000€*	0.73	103 000	0.27

Results derived from 10 000 Monte Carlo simulations. P(CE) denotes the probability that ECPELLA is cost-effective at the specified willingness-to-pay (WTP) threshold based on the net monetary benefit framework. All costs are expressed in 2024€ and discounted at 3% annually together with health outcomes.

PSA, probabilistic sensitivity analysis. Median ICER and probabilities derived from 10 000 Monte Carlo simulations. All values discounted at 3%.

### Deterministic sensitivity analysis

**Figure 4:**
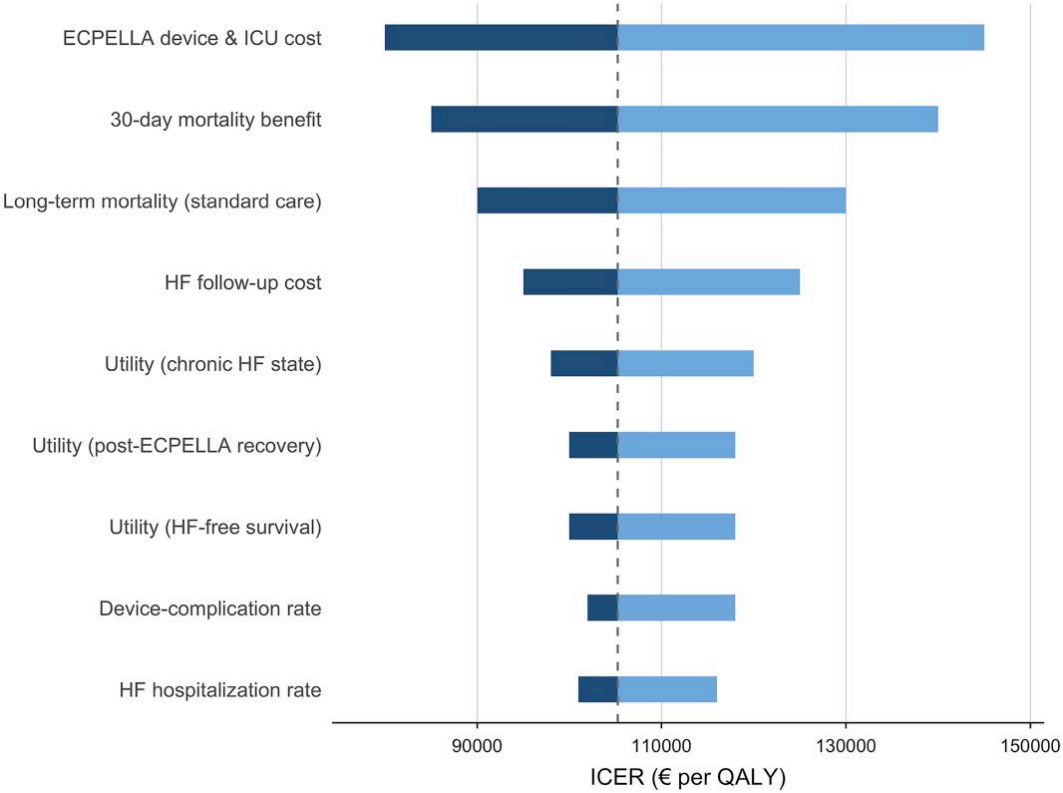
**Tornado diagram for one-way sensitivity analysis of ECPELLA vs. guideline-directed medical therapy (GDMT).** Each horizontal bar represents the impact of varying a single input parameter across its prespecified plausible range on the incremental cost-effectiveness ratio (ICER; € per QALY), holding all other parameters constant. Results correspond to the 10-year base-case analysis with 3% annual discounting of costs and outcomes. The vertical dashed line indicates the base-case ICER (€105 263/QALY). Device acquisition cost, ICU resource use assumptions, and the magnitude of the early mortality effect were the primary drivers of uncertainty, whereas utilities, rehospitalization costs, and complication parameters exerted comparatively smaller influence.

One-way sensitivity analysis showed that cost-effectiveness was most sensitive to the Impella device cost, the assumed ECPELLA mortality hazard ratio, and ICU-day costs (*Figure 4*). Changes in utilities, complication rates, and rehospitalization parameters produced only modest ICER variation. Structural tests, including alternative survival extrapolations, different comparators, and payer vs. societal perspectives, did not materially change the qualitative

conclusion that ECPELLA remained above commonly cited benchmark willingness-to-pay levels.

### Scenario analyses

Scenario analyses showed that cost-effectiveness was strongly dependent on patient characteristics and treatment context. ECPELLA was more favourable in younger patients, reversible aetiologies, early cannulation, and high-volume centres (ICERs €60 000–€80 000/QALY), whereas delayed initiation or absence of early survival benefit produced markedly unfavourable results (>€170 000/QALY). Across all scenarios, ICERs did not fall below commonly cited benchmark WTP levels, confirming the robustness of the base-case conclusion (*Table 4*).

**Table 4:** Deterministic scenario analysis of ECPELLA vs. guideline-directed medical therapy (GDMT)

*Scenario*	ΔCost (€)	ΔQALY	ICER (€/QALY)	P(CE) at €100 000/QALY
*Base case (10-year horizon)*	100 000	0.95	105 263	0.73
*European best-case cost environment (Device −15%, Acute −20%)*	83 000	0.95	87 368	—
*European worst-case cost environment (Device +15%, Acute +20%)*	117 000	0.95	123 158	—
*ECLS–SHOCK-calibrated (neutral early benefit)*	100 000	0.30	333 333	—
*Younger cohort (40–50 years; 30-year horizon)*	110 000	1.80	61 111	—
*Reversible aetiology (myocarditis or pulmonary embolism)*	105 000	1.60	65 625	—
*Delayed cannulation (>24–48 h)*	102 000	0.60	170 000	—
*High-volume centre (>100 ECMO cases/year)*	98 000	1.05	93 333	—
*Low-volume centre (≤50 ECMO cases/year)*	102 000	0.80	127 500	—

**Note**: European transferability scenarios varied device acquisition costs (±15%) and acute-care cost valuation (±20%) to reflect plausible cross-country variation in Western European procurement prices, staffing structures, and ICU unit costs. Incremental health outcomes were held constant.

Incremental costs (ΔCost), incremental quality-adjusted life-years (ΔQALY), and incremental cost-effectiveness ratios (ICER) are shown for the 10-year base-case and predefined structural, clinical, and transferability scenarios. Probability of cost-effectiveness [P(CE)] at a willingness-to-pay threshold of €100 000 per QALY is derived from probabilistic sensitivity analysis where applicable. Costs are reported in 2024€ and discounted at 3% annually together with health outcomes. QALY, quality-adjusted life-year; ICER, incremental cost-effectiveness ratio.

### European transferability scenario analysis (acute-care cost environments)

To assess transferability beyond the German reference costing environment, we performed a deterministic European cost-environment scenario analysis. Incremental effectiveness was held constant (ΔQALY = 0.95), while acute-care cost valuation (±20%) and device procurement prices (±15%) were varied to reflect plausible cross-country variation across Western European health systems.

**Figure 5:**
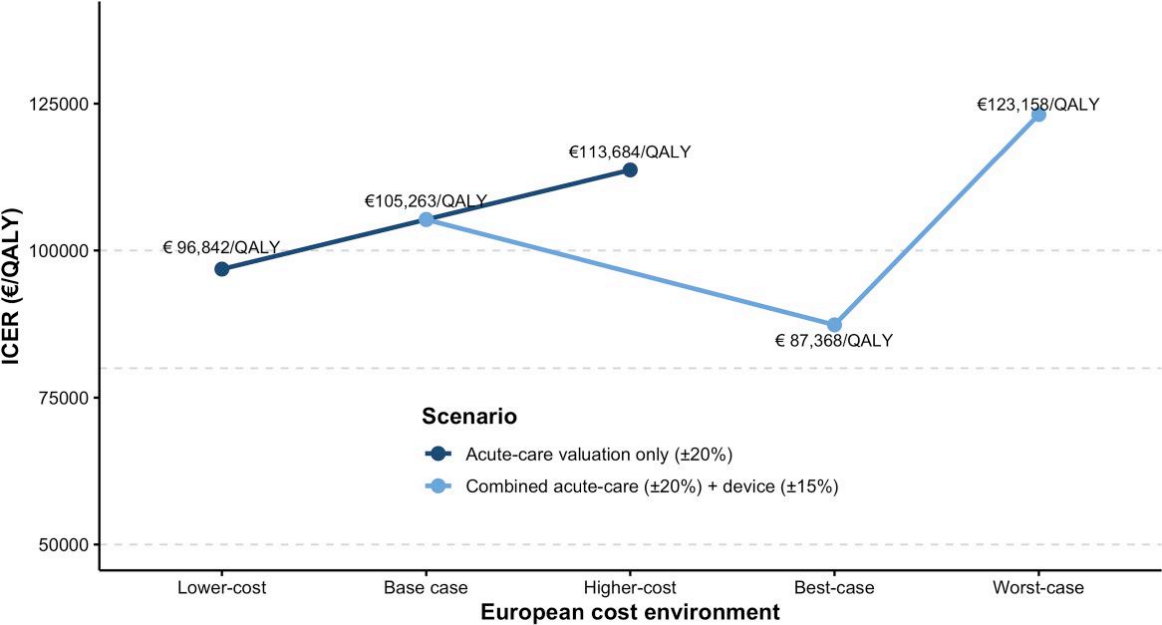
**European transferability scenario analysis.** Incremental cost-effectiveness ratios (ICERs) for ECPELLA vs. guideline-directed medical therapy under alternative Western European cost environments. The dark line shows variation in acute-care cost valuation (±20%), reflecting differences in ICU and ward resource pricing. The light line represents simultaneous variation in acute-care valuation (±20%) and device procurement costs (±15%). Incremental effectiveness was held constant (ΔQALY = 0.95). Dashed horizontal lines indicate commonly cited willingness-to-pay benchmarks (€50 000, €80 000, and €100 000 per QALY). Although absolute ICER values shift with cost structure, the relative economic interpretation remains consistent across plausible European settings.

Varying acute-care costs alone resulted in incremental costs ranging from €92 000 to €108 000 (base case: €100 000), corresponding to ICERs between €96 842/QALY and €113 684/QALY (base case: €105 263/QALY). When device and acute-care costs were varied simultaneously (best- and worst-case European environments), incremental costs ranged from €83 000 to €117 000, yielding ICERs between €87 368/QALY and €123 158/QALY (*Figure 5*).

Using a net monetary benefit framework, INMB remained negative at €50 000/QALY and €80 000/QALY across all modelled European cost environments. At €100 000/QALY, economic neutrality was approached only in the lower-cost scenario and remained negative in higher-cost settings. Across the full range of plausible Western European cost structures, ICERs varied by approximately ±17% around the base-case estimate, but the qualitative inference was unchanged: ECPELLA exceeded commonly cited benchmark willingness-to-pay thresholds in unselected infarct-related CS populations, with more favourable cost-effectiveness confined to predefined clinically selected subgroups and high-volume centres.

### Value-of-information (VOI) analysis

Following ISPOR–SMDM guidance, VOI analysis quantified the expected net monetary consequences of residual parameter uncertainty under the net benefit framework.^40,41^ At a WTP of €80 000 per QALY, the per-patient EVPI was €4 700, increasing to €11 900 at €100 000 per QALY (*Table 5*). These discounted values reflect the maximum amount a decision-maker would be willing to pay to eliminate all parameter uncertainty at the respective WTP levels and are consistent with the remaining decision uncertainty observed in probabilistic analysis. Parameter-specific uncertainty was concentrated in the survival effect and device-related costs.

**Table 5:** Value-of-information (VOI) results per patient

*Willingness-to-pay threshold (€ per QALY)*	*EVPI (€)*	*EVPPI*—*Survival effect (€)*	*EVPPI*—*Device cost (€)*	*Decision uncertainty* [*1* − *P(CE)*]
*80 000*	4700	1200	3500	0.41
*100 000*	11 900	2500	6200	0.27

**Note:** EVPI, expected value of perfect information; EVPPI, expected value of partial perfect information. Values represent the discounted (3% annually) expected net monetary benefit per patient of eliminating overall (EVPI) or parameter-specific (EVPPI) uncertainty. Decision uncertainty is expressed as 1 − P(CE), derived from probabilistic sensitivity analysis. All estimates are based on 10 000 Monte Carlo simulations.

Expected value of partial perfect information values were highest for the ECPELLA mortality hazard ratio and device cost parameters, whereas utilities and rehospitalization costs contributed minimally to overall decision uncertainty (see Supplementary material online, *Figure S3*). Although estimated within the German reference costing framework, VOI results are primarily driven by uncertainty in treatment effectiveness and high-cost resource components. These determinants are not country-specific and are therefore expected to show similar relative importance across comparable Western European health systems operating within similar WTP ranges. Overall, the VOI findings indicate moderate residual uncertainty, with the greatest value of further research arising from improved precision in early survival estimates and device-related cost parameters.

### Policy implications

The sensitivity and value-of-information analyses quantify the implications of residual parameter uncertainty for reimbursement deliberations and research prioritization. Budget impact projections indicate that annual incremental expenditure increases proportionally with higher ECPELLA uptake, reaching approximately €64 million at 40% adoption (see Supplementary material online, *Figure S4*).

Population EVPI and EVPPI provide a monetary measure of the expected benefit of reducing uncertainty under a net monetary benefit framework and can be interpreted alongside the anticipated costs of additional evidence generation. For illustration, an EVPI of €2000 per patient applied to an annual cohort of 500 eligible patients corresponds to a population EVPI of approximately €1 million per year in a mid-sized Western European health system. Parameter-specific EVPPI suggests that uncertainty in early survival benefit and device-related costs contributes most to decision uncertainty. These results describe how decision uncertainty is distributed across model inputs and may inform the prioritization of future data collection and study design.^40,42^

## Discussion

ECPELLA aims to counteract ECMO-related left-ventricular afterload and has been associated with improved short-term outcomes in observational analyses, including lower mortality and enhanced end-organ recovery.^6,7,10^ These potential benefits contrast with increased risks of haemolysis, acute kidney injury, and limb ischaemia and with the substantial resource requirements documented for ECMO and Impella support.^9,10^ This study provides the first structured cost-effectiveness evaluation of ECPELLA vs. GDMT in infarct-related CS from a German payer perspective. ECPELLA improved survival and quality-adjusted life-years but at markedly higher cost, yielding an ICER (€105 263/QALY) above willingness-to-pay ranges commonly applied in Europe. Accordingly, ECPELLA is unlikely to be cost-effective in an unselected CS population. These findings must be interpreted in the context of European care delivery, where ECPELLA use is concentrated in high-volume and university centres, resulting in substantial variation in access and organizational capability across countries.

Across European health systems, the valuation of intensive care resources and device procurement varies. When we modelled plausible lower- and higher-cost European environments, the ICER shifted between approximately €90 000 and €120 000 per QALY, but the overall conclusion remained unchanged. ECPELLA exceeded commonly cited benchmark ranges in unselected CS populations regardless of cost environment. This suggests that economic conclusions are driven more by the magnitude of survival benefit and patient selection than by country-specific reimbursement mechanisms. While absolute ICER values will differ between jurisdictions, across base-case and transferability analyses, ICERs remained above commonly cited benchmark willingness-to-pay values in unselected populations, while ICERs were lower in predefined clinically selected scenarios.

However, more favourable cost-effectiveness estimates were observed in specific clinical subgroups. Subgroups characterized by younger age, reversible aetiologies (e.g. myocarditis or stunned myocardium), early cannulation, and high-volume centres demonstrated ICERs approaching €60 000-80 000/QALY, reflecting higher survival benefit and greater procedural efficiency.^9,19,26^ These gradients mirror real-world variation in outcomes and highlight the importance of structured patient selection and protocolized shock pathways.

The observed subgroup gradients are clinically coherent. In younger patients, early survival gains translate into substantially longer post-discharge life expectancy, allowing the benefit of acute circulatory stabilization to accrue over time. In contrast, in older or highly comorbid individuals, competing mortality risks and limited physiological reserve constrain the durability of short-term haemodynamic rescue, thereby reducing incremental lifetime benefit. Within infarct-related CS, reversibility reflects the extent of salvageable or stunned myocardium following timely revascularization.^3,43^ In patients with preserved myocardial viability, mechanical unloading may facilitate recovery of contractile function and limit adverse remodelling, thereby reducing progression to chronic HF and long-term resource utilization. Under these circumstances, temporary circulatory support functions as a bridge to myocardial recovery rather than to advanced HF.^3,44,45^ Timing and organizational context further modify treatment value. Early cannulation may shorten the duration of systemic hypoperfusion, attenuate multiorgan injury, and preserve renal and neurological function, determinants of whether short-term survival translates into sustained functional recovery.^46^ High-volume centres, supported by structured shock-team pathways and procedural expertise, are more likely to convert acute haemodynamic stabilization into durable clinical benefit rather than prolonged critical illness.^46^

Interpretation of cost-effectiveness in CS must account for remaining life expectancy and long-term recovery potential. Younger patients may derive several additional QALYs from advanced support, justifying higher upfront costs, whereas older or multi-morbid patients achieve limited incremental benefit. If ECPELLA’s true mortality effect were lower, as suggested by neutral findings in ECMO-focused randomized trials and meta-analyses,^17^ both clinical and economic value would decline proportionally. Because early survival benefit represents the principal driver of model outcomes, the economic estimates presented here remain inherently dependent on the magnitude of treatment effect assumed in the model. As further clinical evidence emerges, particularly from randomized or prospective studies, the estimated survival benefit and corresponding economic results may warrant re-evaluation.

Beyond the index hospitalization, a subset of survivors of infarct-related CS progress to advanced HF therapies, including implantable cardioverter-defibrillators or cardiac resynchronization therapy devices (ICD/CRT), durable left-ventricular assist devices (LVAD), or heart transplantation.^27,47^ These interventions are associated with substantial long-term costs but may also confer meaningful gains in survival and health-related quality of life in carefully selected patients.^48–50^ Reliable, comparable estimates of post-shock transition rates to these advanced-therapy pathways were not available for robust incorporation into the present model, particularly in patients treated with ECPELLA. If the modelled early survival benefit associated with ECPELLA translates into greater long-term survivorship, omission of these downstream states may underestimate future costs and modestly overstate cost-effectiveness. Future evaluations should therefore incorporate explicit advanced-therapy health states as longer-term follow-up data and country-specific utilization patterns become available. These longer-term trajectories underscore that the value of advanced circulatory support cannot be judged solely by early survival but must be interpreted within the broader continuum of post-shock care. Decisions regarding escalation to mechanical support therefore intersect not only with acute haemodynamic stabilization but also with downstream resource use, survivorship pathways, and health-system capacity.^43^

Our findings align with contemporary guideline frameworks recommending temporary MCS when pharmacologic therapy fails and emphasizing systems of care, multidisciplinary teams, and early identification of reversible shock.^1,2,16^

By quantifying trade-offs between survival and resource utilization, this analysis contextualizes the economic implications of ECPELLA within contemporary shock care pathways, centre-level capability planning, and reimbursement discussions. A further strength of this evaluation is the integration of real-world comparator data and value-of-information methods, which enhances the interpretability of results across diverse European care settings. Implementation of such guideline-based pathways in Europe is shaped by marked differences in centre volume, resource availability, and shock-team maturity, further amplifying heterogeneity in real-world outcomes and treatment value. The magnitude and direction of our modelled benefits align with observed data showing improved survival when left-ventricular unloading accompanies ECMO and with trial evidence from DanGer-Shock demonstrating mortality benefit with microaxial flow support.^9^ High episode costs for ECMO reported across contemporary registries further support the cost drivers identified in our model.

Ultimately, the value of ECPELLA emerges not solely from its biological effects but from the organizational context in which it is deployed. Centre experience, protocol adherence, and timely escalation strongly influence real-world outcomes.^16^ These system-level determinants are likely to influence real-world outcomes and may explain observed gradients in scenario analyses.^13^ Against this backdrop, our findings provide a quantitative basis to support more consistent, capability-aligned, and equitable use of ECPELLA across European shock systems. Taken together, these findings indicate that the economic value of ECPELLA in infarct-related CS is closely linked to early survival effect and patient selection across high-resource health systems.

### Limitations

This analysis uses contemporary evidence within a transparent partitioned-survival framework and incorporates extensive deterministic, probabilistic, and value-of-information analyses, strengthening confidence in model robustness. The main limitation is that no randomized trial has compared ECPELLA directly with medical therapy; survival and utility inputs therefore rely on indirect or observational data. If the true survival benefit is smaller, or complication rates higher, than assumed, cost-effectiveness would be less favourable. Conversely, lower device or ICU costs or stronger clinical benefit could improve value. Long-term rehabilitation costs and heterogeneity in post-discharge health states were not fully captured, which may underestimate downstream variation. The high uncertainty surrounding short-term survival effects and device-related costs underscores the need for further pragmatic trials and refined cost accounting. The model did not explicitly incorporate downstream advanced HF therapies, such as ICD/CRT implantation, durable LVAD support, or heart transplantation. If ECPELLA increases long-term survival, omission of these health states may underestimate future costs and modestly overstate cost-effectiveness.

## Conclusion

In this model-based analysis, ECPELLA was associated with increased survival and quality-adjusted life-years at substantially higher cost. The base-case ICER was €105 263 per QALY. More favourable cost-effectiveness estimates were observed in predefined scenario analyses involving younger age, potentially reversible myocardial dysfunction, early cannulation, and treatment in high-volume centres. Results were sensitive to assumptions regarding treatment effect and device-related costs. Residual decision uncertainty suggests that further prospective evidence could meaningfully influence economic estimates.

## Supplementary Material

oeag064_Supplementary_Data

## Data Availability

This analysis was based exclusively on aggregated data derived from previously published studies and publicly available sources cited in the manuscript. No individual participant data were collected or generated for this study. All model input parameters, assumptions, and supplementary material online, tables supporting the findings of this analysis are available from the corresponding author upon reasonable request.

## Supplementary material

Supplementary material is available online and includes detailed model assumptions, input parameters, calibration procedures, sensitivity analyses, validation procedures, and the CHEERS 2022 checklist.
