# Supplementary material for: Cost-effectiveness of combined veno-arterial extracorporeal membrane oxygenation and Impella support (ECPELLA) in infarct-related cardiogenic shock
Source: Eur Heart J Open. 2026 Apr 15;6(2):oeag064. doi: 10.1093/ehjopen/oeag064 (PMC13162026; doi:10.1093/ehjopen/oeag064)
Supplement: oeag064_Supplementary_Data [file oeag064_supplementary_data.docx]

# **Supplementary Material**

**Supplementary Methods**

## **Model structure and analytic framework**

A partitioned-survival model (PSM) was used to evaluate combined veno-arterial extracorporeal membrane oxygenation and microaxial left ventricular unloading (ECPELLA) versus guideline-directed medical therapy (GDMT) in infarct-related cardiogenic shock (CS). Monthly cycles were applied during the first year and annual cycles thereafter. Three health states were modelled: alive without heart failure (HF), alive with HF, and dead. Time spent in each state was derived from the area under the overall-survival and HF-free survival curves. A 10-year analytic horizon and a 3% annual discount rate were applied.

**Derivation of clinical effectiveness inputs and survival calibration**

To provide clinical transparency regarding how survival assumptions were constructed, we outline below the data sources and modelling steps used to derive baseline GDMT survival and to apply the ECPELLA treatment effect.

*Baseline GDMT survival*

Baseline overall survival under GDMT was constructed from contemporary randomized and registry data reflecting modern revascularization and ICU practice. The primary data sources were: The control arm of ECLS-SHOCK [1], The control arm of ECMO-CS [2], Contemporary CS registries including Lang et al. 2021, Morrow et al. 2024 (AHA registry), and Lauridsen et al. 2021 [3-5]. Kaplan–Meier survival curves were digitized and fitted using parametric survival models (Weibull, Gompertz, log-logistic, and flexible Royston–Parmar models). Model selection was based on Akaike and Bayesian information criteria, visual goodness-of-fit, and clinical plausibility of long-term extrapolation. External calibration ensured alignment with observed mortality anchors of approximately: 50% at 30 days and 40–45% at 1 year in contemporary PCI-treated CS cohorts [1, 2, 4] (Table S0).

*Application of ECPELLA treatment effect*

In the absence of a direct randomized ECPELLA vs GDMT comparison, the relative mortality reduction observed in the DanGer-Shock trial [6] (HR 0.74, 95% CI 0.55–0.99) was applied to the calibrated GDMT baseline survival curve under a proportional hazards assumption. No independent mortality benefit was assumed for ECMO alone, consistent with the neutral findings of ECLS-SHOCK.

*Role of observational ECPELLA data*

Observational ECPELLA registry studies [7-10] were used to contextualize plausibility of short-term mortality reductions and were explored in scenario analyses. They were not used to parameterize the base-case treatment effect to avoid potential confounding and double counting of survival benefit.

**Table S0. Sources and role of survival inputs in the base-case model**

| Component | Data Source | Time Points Used | Role in Model |
| --- | --- | --- | --- |
| Baseline GDMT survival | ECLS-SHOCK (control arm) [1] | 30d, 180d | Primary anchor for early survival |
| Baseline GDMT survival | ECMO-CS (control arm) [2] | 30d | Cross-validation of short-term survival |
| Registry validation | Lang et al. 2021 [3] | 1y | External validation of mid-term survival |
| Registry validation | Morrow et al. 2024 [4] | 1y | External validation |
| Long-term plausibility | Lauridsen 2021 [5] | 3–5y | Long-term survival plausibility |
| Relative treatment effect | DanGer-Shock [6] | 180d | Base-case hazard ratio (HR 0.74) |
| Observational ECPELLA | Schrage 2020 [9]; Patel 2019 [7]; Bhatia 2022 [8]; Bogerd 2023 [10] | 30d | Scenario and plausibility analyses only |

**Implementation of survival functions in the model**

The calibrated GDMT baseline survival function served as the reference trajectory for all subsequent projections. ECPELLA survival was derived by applying the DanGer-Shock hazard ratio (HR 0.74) under a proportional hazards assumption, such that:

$$S_{\text{ECPELLA}}(t)=S_{\text{GDMT}}(t)^{HR}$$

This approach ensures that the relative treatment effect is consistently applied over time while preserving the empirically calibrated baseline mortality structure.

Beyond the observed follow-up period of the informing trials, long-term survival was extrapolated using the selected parametric model while maintaining excess mortality relative to age-matched general population life tables. Alternative extrapolation functions and neutral survival scenarios were evaluated in structural sensitivity analyses to assess robustness.

***Heart-failure incidence***

Incident HF following CS was estimated using longitudinal registry data comparing post-shock and post-myocardial infarction outcomes [5, 11]. This study reported a substantially increased long-term risk of HF hospitalization among shock survivors (HR ≈ 2.9), which informed the annual transition probability to the HF health state in the model. Once patients entered the HF state, they remained in that partition, reflecting the chronic and progressive nature of post-shock ventricular dysfunction, with mortality governed by the overall survival function.

***Health-state utilities***

Health-state utilities were specified on a 0–1 scale and informed by EQ-5D–based assessments in CS and post–myocardial infarction populations.

For survivors without chronic HF, a base-case utility of 0.80 was assigned, representing clinically stable post-infarction recovery following successful revascularization and shock resolution [12]. For patients who developed chronic HF (NYHA class II–III), a lower utility of 0.65 was assigned to reflect persistent symptom burden and functional limitation. This assumption is consistent with mid-term follow-up data demonstrating incomplete functional recovery after severe CS, including cohorts achieving native heart recovery following Impella 5+ support [13].

To account for early morbidity associated with CS and temporary mechanical circulatory support, a short-term utility decrement of −0.10 was applied during the acute post-shock phase (0–6 months). This assumption is supported by prospective studies of VA-ECMO survivors reporting that although many patients achieve favorable functional outcomes, a clinically relevant subset experience persistent limitations in mobility, physical function, and mental health domains [14, 15]. These findings support modelling a temporary reduction in early post-ICU health utility.

Utilities were valued using the EQ-5D-5L value set corresponding to the reference costing environment. In probabilistic sensitivity analyses, utilities were modelled using beta distributions to reflect uncertainty within the bounded 0–1 scale [16, 17].

**Costs (payer perspective, 2024 €)**

Costs were estimated from a statutory health insurance payer perspective, using the German DRG-based framework as the reference costing environment. Cost components comprised device acquisition (Impella and VA-ECMO), ICU and ward length of stay, revascularization procedures, management of device-related complications, diagnostics, and long-term HF care including rehospitalizations. Device and hospitalization costs were derived from German DRG tariffs and institutional micro-costing data and cross-validated against published European cost analyses [18-20]. All costs were standardized to 2024 €.

Costs were modelled using gamma distributions in probabilistic analyses. Deterministic sensitivity analyses varied key cost drivers, particularly device acquisition and ICU length of stay, across plausible ranges.

**Economic outcome definitions**

The model estimated total costs, life-years (LYs), QALYs, and incremental cost (ΔCost) and effectiveness (ΔQALY).

$$ICER=\frac{\Delta Cost}{\Delta QALY}$$

Incremental net monetary benefit (INMB) was calculated across willingness-to-pay (WTP) thresholds (€30,000–€100,000/QALY):

$$INMB=\lambda\times\Delta QALY-\Delta Cost$$

A positive INMB indicates that ECPELLA is cost-effective at the specified willingness-to-pay value (λ) [21].

**Uncertainty analysis**

Parameter uncertainty was explored via probabilistic sensitivity analysis (PSA) using 10,000 Monte-Carlo simulations, applying beta distributions for probabilities and utilities, gamma for costs, and log-normal for hazard ratios. One-way sensitivity analyses varied each parameter across 95% confidence bounds or plausible ranges. Scenario analyses examined alternative ages, etiologies, initiation timings, and center volumes.

*Probabilistic sensitivity analysis (PSA)*

A 10 000-iteration Monte-Carlo simulation jointly sampled all model parameters. Correlation between selected parameters was incorporated where supported by data; otherwise, parameters were sampled independently. Results were summarized on the cost-effectiveness plane and expressed as a cost-effectiveness acceptability curve (CEAC) [22]. Convergence was verified by stability of mean ICER values after 5 000 iterations.

*Deterministic sensitivity analysis*

A one-way sensitivity analysis (OWSA) varied each parameter over its 95 % confidence interval or ± 30–50 % range. Results were displayed as a tornado diagram highlighting the most influential parameters, including the Impella hazard ratio, ICU-day cost, and device price. Structural sensitivity analyses tested alternative survival extrapolations and a scenario using a conventional three-state Markov structure (Alive without HF / HF / Dead) for comparison.

*Value-of-information (VOI) analysis*

A value-of-information (VOI) framework was applied to quantify the impact of parameter uncertainty on decision-making, following ISPOR–SMDM good practice recommendations [23, 24]. The expected value of perfect information (EVPI) was estimated across WTP thresholds (€30 000–€100 000 per QALY), representing the potential benefit of completely eliminating all parameter uncertainty [25, 26]. In addition, the expected value of partial perfect information (EVPPI) was computed for grouped parameters—specifically the survival hazard ratio of ECPELLA, device and ICU costs, and utility values. EVPPI quantifies the potential value of additional research targeting specific uncertain inputs. A high EVPI indicates that residual uncertainty could alter reimbursement decisions and thus justifies further evidence generation, whereas a high EVPPI identifies the parameters contributing most to overall decision uncertainty [27].

**Model validation and transparency**

**Model validation was conducted in accordance with established ISPOR–SMDM good research practice recommendations for decision-analytic modelling to ensure face validity, internal consistency, structural robustness, and external credibility of the analytical framework** [28-34].

***Face validity***

**The overall model structure, clinical assumptions, and treatment pathways were reviewed by an interdisciplinary expert panel comprising intensivists, a cardiac surgeon, and health economists with expertise in CS management and health-economic evaluation. The panel confirmed that the health states, transition logic, resource use assumptions, and outcome definitions reflected contemporary clinical practice and current standards of advanced circulatory support.**

***Internal validity***

**Internal validity was assessed through structured logical consistency checks and extreme-value testing. Key parameters were varied to implausible boundary values (e.g., zero device cost, neutral treatment effect) to verify directional responsiveness of incremental costs and QALYs. Deterministic base-case results were cross-checked against probabilistic simulation means to confirm numerical stability and absence of programming artefacts. Model outputs were independently reproduced using fixed random-number seeds to ensure computational reproducibility.**

***Structural validation***

**Structural robustness was examined using a simplified state-transition (Markov) validation model employing identical clinical inputs and time horizons. Cost and QALY estimates from this alternative framework differed by less than ±5% from those of the primary partitioned-survival model, supporting structural consistency across modelling approaches.**

***External calibration and validation***

**Model-predicted short- and mid-term survival outcomes were calibrated against contemporary randomized trials and multinational CS registries. Predicted 30-day, 1-year, and 3-year survival estimates aligned within ±5% absolute difference of observed reference data. Parametric survival extrapolations (Weibull and Gompertz distributions) were selected based on Akaike and Bayesian information criteria and visual inspection to ensure clinically plausible long-term trajectories, including maintenance of excess mortality relative to age-matched population life tables.**

***Transparency and reproducibility***

**All model construction, parameterization, probabilistic sensitivity analyses, and value-of-information calculations were performed in R version 4.3 (R Foundation for Statistical Computing, Vienna, Austria). Annotated code, parameter distributions, and full input tables are provided in the Online Appendix and are available upon reasonable academic request. Reporting adheres to ISPOR modelling standards and CHEERS 2022 guidelines.**

## **Clinical inputs and transition formulas**

To support structural validation of the partitioned-survival model, we implemented an auxiliary state-transition (Markov) validation model using identical clinical inputs, time horizons, and discounting assumptions as the base-case analysis. The model comprised three mutually exclusive health states (Alive without HF, Alive with HF, Dead) and was used to verify transition probability implementation and internal consistency of cost and QALY accumulation. The state-transition structure is shown in Figure S1.

***Event definitions***

Clinical events were defined according to contemporary registry and trial standards. Device-related complications included **major bleeding** (BARC ≥ 3), **limb ischemia**, **hemolysis, stroke or transient ischemic attack (TIA),** and **infection**. These events were incorporated into the cost structure and utility decrements but did not constitute separate health states.

***Conversion of 30-day to monthly transition probabilities***

Transition hazards were converted from published 30-day risks using standard exponential transformation formulas:

$$h=-\ln(1-p_{30d})/30;p_{1m}=1-e^{-h\times30}.$$

The **acute phase (0–6 months)** used phase-specific hazards reflecting early post-shock risk, whereas the **chronic phase (>6 months)** applied constant hazards.

***Rehospitalization***

Monthly rehospitalization probabilities were derived from multicenter **HF and CS registries**, with competing-risk transitions to death. These probabilities informed both direct hospitalization costs and reductions in quality-adjusted life-years (QALYs).

***Long-term survival***

Parametric survival functions **(Weibull, Gompertz,** and **flexible parametric spline models)** [35] were fitted to long-term follow-up data from shock registries. The best-fit distribution was selected based on Akaike and Bayesian information criteria and calibrated so that predicted **1-year and 3-year survival** matched observed registry data within ± 5 % absolute error.

***Model parameters***

Key model input parameters—including base-case values, uncertainty ranges, and distributions used for one-way and probabilistic sensitivity analyses—are summarized in **Table S1-S2**. Utility values and temporary decrements applied to health states and adverse events are reported in **Table S3**. These inputs were derived from published literature, registry sources, and institutional costing data, adjusted to 2024 € and discounted at 3 % annually according to national guidelines.

**Supplementary Table S1. ICU per-diem micro-costing decomposition (reference setting) and linkage to Western European benchmarking (2024 €)** The base-case model applies an average ICU per-diem of €2,000 (see Table S2). For transparency, this per-diem is decomposed into staffing, drugs and medicinal products, infrastructure/overhead, diagnostics, and consumables using German ICU cost-unit accounting and national cost analyses (Moerer et al., 2007; Martin et al., 2008). The components sum to the aggregated ICU per-diem and are not modelled as separate additive costs in the base-case analysis. The decomposition reflects the mean resource composition of contemporary ICU care and does not imply uniform cost allocation at the individual patient level **[11, 36-41]**.

**A ICU per-diem decomposition (base ICU day; sums to €2,000)** **[42, 43]** The base-case model applies an aggregated ICU per-diem of €2,000. The decomposition below reflects Wester European ICU cost-unit accounting and costing evidence and is provided for transparency. The components sum to the aggregated per-diem and are not implemented as separate additive costs in the model.

| ICU cost component (per ICU day) | Evidence anchor (Germany) | Share of total ICU per-diem | Value if ICU per-diem = €2,000 |
| --- | --- | --- | --- |
| Staffing (nursing + physicians) | Personnel is the dominant cost driver in European ICU costing | **53%** | **€1,060** |
| Drugs + medicinal products (incl. vasoactives/inotropes, sedation/analgesia, antibiotics, anticoagulation, fluids) | Drugs/medicinal products constitute a major non-personnel share | **18%** | **€360** |
| Infrastructure/overhead (space, equipment depreciation, administration, utilities) | Infrastructure/overhead is a stable contributor in European cost-unit accounting | **16%** | **€320** |
| Diagnostics & support services (labs, blood gases, imaging allocation) | Remaining components captured in European ICU micro-costing frameworks | **8%** | **€160** |
| Consumables (non-device disposables) (lines, tubing, PPE, routine supplies) | Remaining components captured in European ICU micro-costing frameworks | **5%** | **€100** |
| Total ICU per-diem | — | **100%** | **€2,000** |

**B Resource-intensity add-ons [44, 45]** Incremental daily costs associated with high-intensity ICU care are reported below for transparency [49,50]. These estimates reflect additional resource use attributable to invasive mechanical ventilation and continuous renal replacement therapy compared with standard ICU management. They are not applied in the base-case per-diem calculation but were evaluated in sensitivity analyses to assess the impact of variability in organ-support intensity.

| Resource-intensity factor | Incremental cost (€/day) | Range (OWSA) | Distribution | Source |
| --- | --- | --- | --- | --- |
| Invasive mechanical ventilation | +€600 | €300–€900 | Gamma | Kaier et al., 2020 [44] |
| Continuous renal replacement therapy (CRRT) | +€300 | €100–€1,000 | Gamma | Srisawat et al., 2010 [45] |

**C Device costs (additive; per patient/episode; applied additively in model as in Table S2)**

| Device | Base (€) | Range (OWSA) | Distribution | Source |
| --- | --- | --- | --- | --- |
| Impella device + technical | 57,770 | 45,000–65,000 | Gamma | Rognoni et al., 2025 [46] |
| VA-ECMO episode | 60,000 | 50,000–75,000 | Gamma | Mishra et al., 2010; Oude Lansink-Hartgring et al., 2021; institutional costing [19, 20] |

**Figure S1. Model structure for the ECPELLA cost-effectiveness analysis.** Three health states—Alive without HF, Alive with HF, Dead. Cycle length one month; transitions parameterized from registry and trial data.


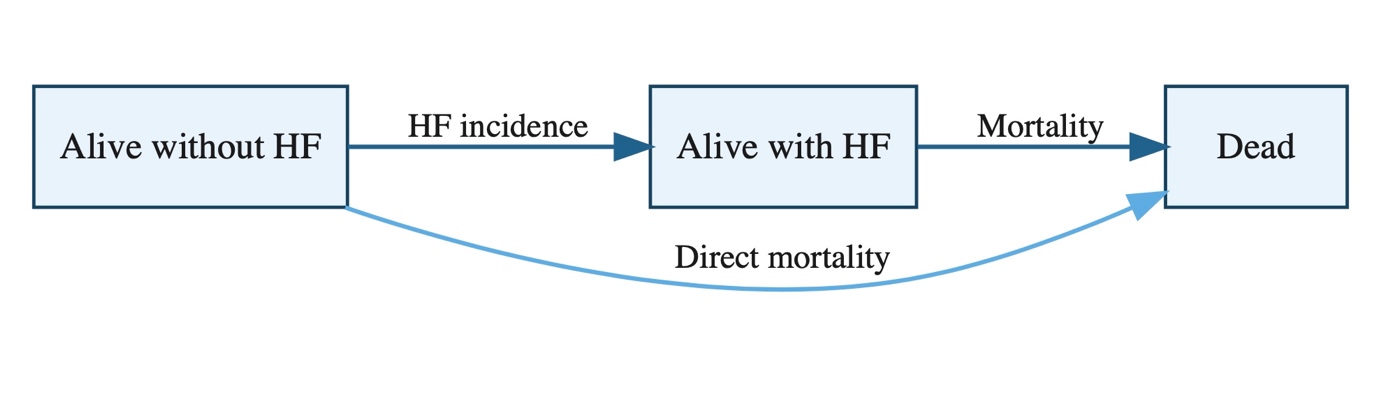


**Table S2. Key model input parameters and Western European benchmarking ranges (base values, deterministic ranges, PSA distributions, and sources)**

| Parameter | Base | Range (OWSA) | PSA Distribution | Source / Note |
| --- | --- | --- | --- | --- |
| ***Clinical parameters*** |  |  |  |  |
| GDMT 30-day mortality | 0.50 | 0.40–0.60 | Beta | Calibrated to contemporary CS RCT and registry data (Thiele et al., 2023; Lang et al., 2021; Morrow et al., 2024) [1, 3, 4, 47] |
| ECPELLA vs GDMT HR (mortality) | 0.74 | 0.55–0.99 | Log-normal | DanGer-Shock trial[6] |
| Annual post-discharge mortality (GDMT) | 0.15 | 0.10–0.20 | Beta | Contemporary CS registry extrapolation [3, 4] |
| Annual HF incidence | 0.08 | 0.05–0.12 | Beta | Post-CS HF risk [48, 49] |
| ***Utilities (EQ-5D based)*** |  |  |  |  |
| Utility (post-shock without HF) | 0.80 | 0.75–0.85 | Beta | EQ-5D values in CS/HF populations [12, 50] |
| Utility (with HF, NYHA II–III) | 0.65 | 0.60–0.70 | Beta | EQ-5D values in HF [12, 50] |
| Acute disutility (0–6 months) | −0.10 | −0.05 to −0.15 | Bounded decrement | Post-ICU morbidity and quality-of-life impairment [12] |
| ***Acute hospitalization costs (2024 €, GKV perspective)*** | | | | |
| Impella device + technical cost | €57,770 | €45,000–65,000 | Gamma | European DRG mapping; cost-effectiveness analyses [46] |
| VA-ECMO device + setup (excluding ICU days) | €40,000 | €30,000–55,000 | Gamma | European ECMO micro-costing studies [19, 20] |
| ICU day cost (GDMT) | €2,000 | €1,500–3,000 | Gamma | Western European ICU cost-unit accounting [42, 43] |
| ICU intensity multiplier (ECPELLA vs GDMT) | 1.30 | 1.10–1.60 | Log-normal | Reflects higher organ support burden under ECPELLA [11, 51] |
| Mean ICU LOS (GDMT) | 6 days | 4–10 days | Gamma | Contemporary CS cohorts [3, 47] |
| Mean ICU LOS (ECPELLA) | 12 days | 8–18 days | Gamma | Increased resource intensity under device support [6, 51] |
| Ward day cost | €600 | €400–900 | Gamma | Western European [42, 43] |
| Mean ward LOS | 6 days | 4–12 days | Gamma | Derived from trial hospitalization data [6] |
| PCI / revascularization cost | €5,000 | €4,000–10,000 | Gamma | Western European reimbursement [40, 41, 52] |
| Major bleeding (per event) | €5,000 | €3,000–8,000 | Gamma | Acute management cost [53] |
| Limb ischemia (per event) | €7,000 | €4,000–10,000 | Gamma | Vascular intervention costs[43, 53] |
| Stroke (acute phase) | €10,000 | €6,000–15,000 | Gamma | Acute inpatient cost estimates [54, 55] |
| Sepsis (per event) | €6,000 | €3,000–10,000 | Gamma | ICU infection-related cost increment [56] |
| ***Long-term costs*** |  |  |  |  |
| Annual HF management (≥ Year 2) | €3,000 | €1,000–5,000 | Gamma | European HF cost studies [54, 55] |
| HF rehospitalization (per event) | €5,000 | €3,000–7,000 | Gamma | European HF registry and payer data [57, 58] |
| **Economic parameters** |  |  |  |  |
| Discount rate | 3% | 0–5% | Fixed | European health-economic guidance [23, 59] |
| Currency year | 2024 € | — | — | Standardized using German health-sector inflation indices |

***Note****: Where available, deterministic ranges were selected to reflect variability reported in Western European ICU cost-unit accounting and micro-costing studies (including ECMO and organ-support costs). This benchmarking was used to assess external plausibility across comparable high-income health systems; base-case costing remains referenced to the German statutory health insurance setting. ICU per-diem cost composition is reported in Table S8.*

**Table S3. Utilities (EQ-5D-5L) and temporary decrements**

| Health state / event | Base utility / decrement | Range (OWSA) | Distribution (PSA) | Duration / application rule | Notes |
| --- | --- | --- | --- | --- | --- |
| Post-shock without HF | 0.80 | 0.75–0.85 | Beta | Applied per cycle while in “Alive without HF” state | Reflects stable post-infarction recovery |
| Post-shock with HF (NYHA II–III) | 0.65 | 0.60–0.70 | Beta | Applied per cycle while in “Alive with HF” state | Persistent functional limitation |
| Acute post-shock disutility | −0.10 | −0.05 to −0.15 | Bounded decrement | Applied during first 6 months only | Captures post-ICU syndrome and early morbidity |
| Rehospitalization (HF) | −0.15 | −0.10 to −0.20 | Bounded decrement | Applied for one model cycle | Additive to underlying state utility |
| Device-related complication | −0.25 | −0.15 to −0.30 | Bounded decrement | Applied for one model cycle | Applied additively; bounded at utility ≥ 0 |

**Scenario and subgroup analyses**

Deterministic scenario analyses were conducted to explore structural and clinical uncertainty in the base-case model. Each scenario varied predefined key parameters while maintaining all other assumptions constant; all results were discounted at 3% per annum. The one-way sensitivity analysis evaluated the impact of parameter variation on the ICER, as shown in Figure 3. Changes in post-ECPELLA utility, rehospitalization rates, and device-related complication costs had comparatively smaller effects, indicating that cost-effectiveness was primarily driven by survival assumptions and device- and ICU-related costs.

***Scenario definitions***

The **base-case** represented a 60-year-old patient cohort treated with ECPELLA compared to GDMT over a 10-year time horizon. Alternative scenarios assessed plausible variations in patient characteristics and treatment conditions:

- **ECLS-SHOCK-like**: Assumed neutral early mortality benefit, reflecting results of contemporary randomized evidence.
- **Younger cohort (30-year horizon)**: Tested long-term cost-effectiveness in patients with greater residual life expectancy.
- **Reversible etiology**: Represented patients with potentially transient causes of shock (e.g., myocarditis, stunned myocardium).
- **Delayed initiation (>24 h)**: Simulated late ECPELLA deployment relative to shock onset.
- **High-volume center**: Modelled lower per-procedure costs and improved outcomes observed in experienced institutions.

**Probabilistic sensitivity analysis (PSA)**

Parameter uncertainty was addressed using PSA with 10,000 Monte Carlo simulations. Probability and utility parameters were modelled using beta distributions, cost parameters using gamma distributions, and relative treatment effects (hazard ratios) using log-normal distributions. All parameters were sampled jointly in each iteration to propagate uncertainty through the model. Correlated inputs (e.g., length of stay and cost) were handled using Cholesky decomposition where appropriate. All costs and outcomes were discounted at 3% annually.

**Supplementary Results**

*Results of probabilistic sensitivity analysis*

Across 10,000 simulations, the mean incremental cost of ECPELLA versus GDMT was €100,000 (95% credible interval €20,000–€180,000), and the mean incremental QALY gain was 0.95 (0.10–1.80). The corresponding mean ICER was €105,000 per QALY gained, with a right-skewed distribution (median €103,100 per QALY). Incremental net monetary benefit (INMB) remained negative at a WTP threshold of €50,000 per QALY and approached INMB remained negative at λ ≤ €100,000/QALY. All simulations fell within the northeast quadrant of the cost-effectiveness plane, indicating that ECPELLA was consistently more effective but more costly than GDMT (Table S4).

**Table S4. Probabilistic Sensitivity Analysis (10,000 Monte Carlo simulations; 3% discounting)**

| Metric | Mean | Median | 2.5 % | 97.5 % |
| --- | --- | --- | --- | --- |
| Incremental cost (ΔCost, €) | 100,000 | 98,200 | 20,000 | 180,000 |
| Incremental QALYs (ΔQALY) | 0.95 | 0.92 | 0.10 | 1.80 |
| ICER (€/QALY) | 105,000 | 103,100 | 60,000 | 300,000 |
| Incremental net monetary benefit (INMB) @ €50,000/QALY (€) | −52,500 | −50,000 | −120,000 | 15,000 |
| Incremental net monetary benefit (INMB) @ €100,000/QALY (€) | −5,000 | −3,800 | −80,000 | 60,000 |

***Note:*** *Credible intervals reflect the 2.5th and 97.5th percentiles of the simulated distributions. Because ICERs are ratios of two stochastic quantities, interval estimates are reported descriptively; decision uncertainty is primarily interpreted using the net monetary benefit framework.*

**Cost-effectiveness acceptability curve**

The cost-effectiveness acceptability curve (CEAC) presents the probability that ECPELLA is cost-effective across alternative WTP thresholds. The probability of cost-effectiveness increased with higher WTP values, reaching 10% at €30,000/QALY, 20% at €50,000/QALY, 59% at €80,000/QALY, and 73% at €100,000/QALY. The distribution of simulated ICERs was right-skewed, with median values ranging between approximately €103,000 and €105,000 per QALY.

**Deterministic sensitivity analysis**

We also tested the inclusion or exclusion of rare long-term adverse event costs. These analyses follow NICE recommendations (updated 14 July 2025) to explore uncertainty in extrapolation [60, 61], care pathways, and perspective assumptions. None of these alternative scenarios materially changed the qualitative conclusion that ECPELLA’s ICER remained above commonly cited benchmark WTP ranges. For transparency, the ranges explored in the tornado analysis included:

- Device and ICU costs (±25–50 % of base value)
- Complication rates (95 % CI from clinical trials)
- Survival benefit (± CI from meta-analytic data)
- Health-state utility values (± plausible bounds or 95 % CI)

Illustratively, halving the incremental survival benefit increased the ICER to approximately €210 000 per QALY, whereas doubling it reduced the ICER to €53 000 per QALY. The complete results are presented in the accompanying tornado diagram.

**Net monetary benefit and value-of-information analyses**

The net monetary benefit (NMB) approach was used to translate incremental costs and QALYs into a common monetary metric across alternative WTP thresholds. INMB was calculated as:

$$\text{INMB}=(\lambda\times\Delta\text{QALY})-\Delta\text{Cost}$$

where λ denotes the societal WTP per QALY. With base-case incremental cost fixed at €100 000 and incremental effectiveness of 0.95 QALY, deterministic INMB values remain negative below a WTP of approximately €105 000/QALY (Table S5). At λ = €100 000, the model approaches economic neutrality, consistent with the observed probability of cost-effectiveness of ≈ 0.44 in probabilistic analysis.

*Incremental NMB at different WTP thresholds*

Table S6 presents the INMB of ECPELLA versus GDMT across commonly applied WTP thresholds in cardiovascular health economics. Calculations are based on the base-case incremental cost of €100 000 and incremental effectiveness of 0.95 QALYs. The INMB remains negative across all thresholds below €100 000 per QALY, indicating that ECPELLA is not cost-effective at commonly cited benchmark willingness-to-pay values (€30 000–€50 000 per QALY). Economic neutrality (INMB = 0) is reached only near a threshold of approximately €105 000 per QALY (Table S6).

**Table S5. Deterministic incremental net monetary benefit (INMB) across willingness-to-pay thresholds (base-case values)**

| WTP (€/QALY) | INMB per patient (€) | Probability cost-effective (%) | Decision status |
| --- | --- | --- | --- |
| 30,000 | −71,500 | 10 | INMB < 0 |
| 50,000 | −52,500 | 20 | INMB < 0 |
| 80,000 | −24,000 | 59 | INMB < 0 |
| 100,000 | −5,000 | 73 | INMB < 0 |

***Note:*** *Deterministic incremental net monetary benefit (INMB) was calculated as INMB = λ × ΔQALY – Δcost using base-case values (ΔCost = €100,000; ΔQALY = 0.95). Probability of cost-effectiveness reflects probabilistic sensitivity analysis results at each willingness-to-pay (λ) value. Negative INMB values indicate that incremental costs exceed monetized incremental health benefits at the specified λ.*

*INMB across WTP thresholds and ΔQALY with ΔCost fixed at €100,000 (illustrative)*

The INMB surface visualizes the interaction between WTP (x-axis) and incremental QALYs (y-axis) for ECPELLA versus GDMT, assuming ΔCost = €100 000. The yellow contour indicates the break-even frontier (INMB = 0), corresponding to ≈ 1.0 QALY at λ = €100 000, consistent with deterministic thresholds in Table S5. Positive (green–yellow) regions represent combinations where ECPELLA becomes cost-effective, whereas negative (blue–purple) regions denote economic dominance by GDMT. Across plausible WTP ranges (λ = €30 000–€100 000/QALY), INMB increases linearly with incremental QALYs, reflecting the steep cost gradient of mechanical circulatory support (Figure S2).

**Figure S2. INMB** Surface incremental net monetary benefit (INMB) surface for ECPELLA versus GDMT. ΔCost was fixed at €100,000, and WTP ranged from €20,000 to €140,000 per QALY. The yellow contour indicates the break-even frontier (INMB = 0), corresponding to approximately one incremental QALY at a WTP of €100,000, consistent with deterministic NMB thresholds reported in Table S5.


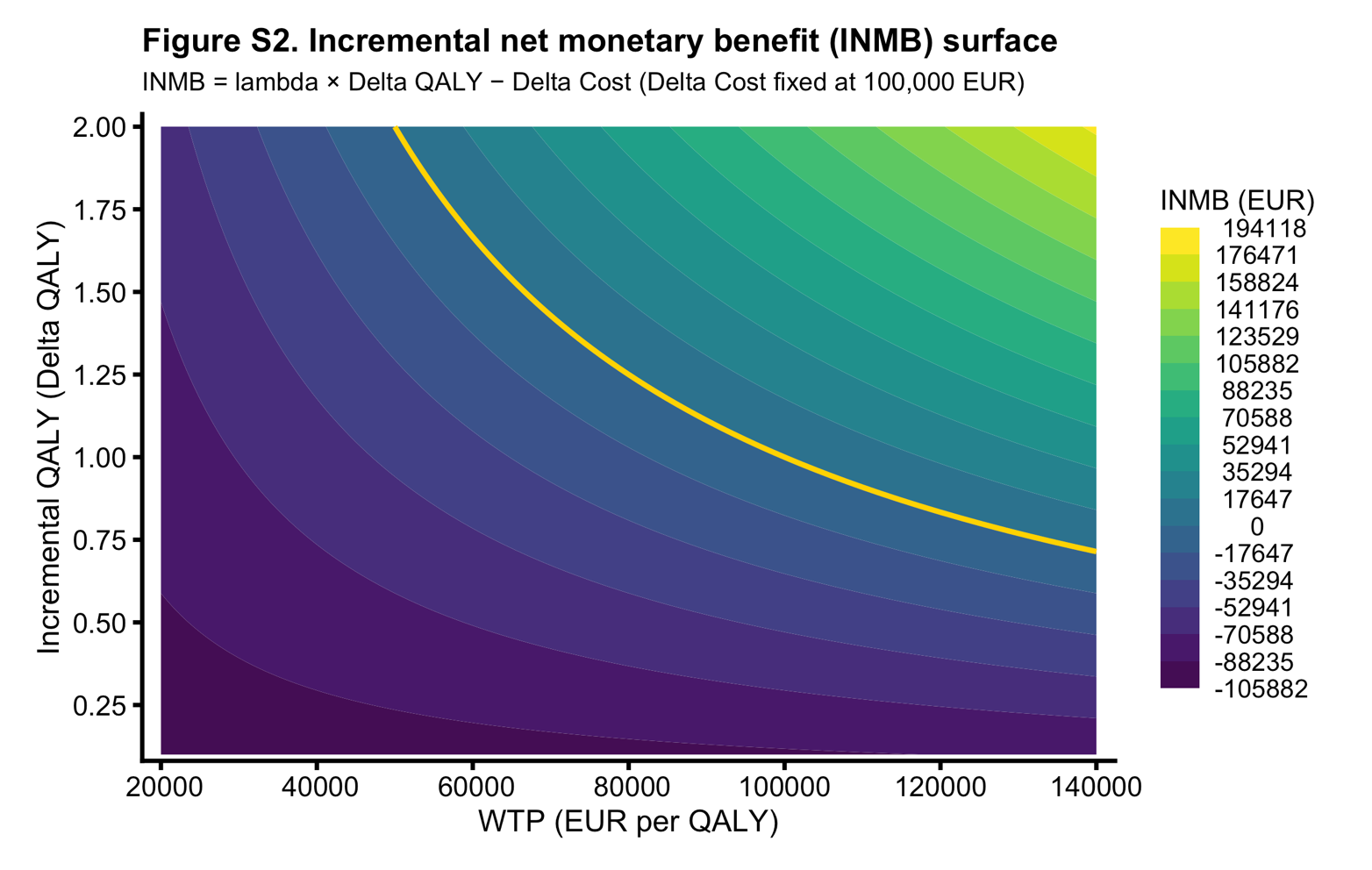


Deterministic and probabilistic results jointly indicate that ECPELLA would become cost-effective only under scenarios with substantial survival gains (> 1.0 QALY) or device/ICU cost reductions exceeding 40–50 %. Subsequent value-of-information (VOI) analysis (Figure S3) quantified residual decision uncertainty across parameter groups, identifying survival benefit and device cost as the most influential sources of uncertainty (EVPPI € 2 500 and € 6 200 per patient, respectively).

**Scenario analysis**

Scenario analyses were performed to examine structural and parameter uncertainty under extreme but plausible parameter combinations. The best-case scenario combined higher survival benefit estimates, improved post-shock utility values, and lower device and acute-care costs. The worst-case scenario incorporated lower treatment effectiveness, reduced utility values, and increased acute-care resource use.

Under best-case assumptions, incremental QALYs increased and incremental costs decreased relative to the base case, resulting in a lower ICER compared with the base-case estimate. Under worst-case assumptions, incremental effectiveness decreased and total costs increased, resulting in a substantially higher ICER, and in some simulations cost-dominance of GDMT (higher cost and lower effectiveness for ECPELLA).

Across all tested parameter combinations, ICER variation remained primarily driven by survival assumptions and device-related cost inputs. The predefined European cost-environment scenario (±20% acute-care valuation; ±15% device procurement costs) altered incremental costs but did not change incremental effectiveness. Under these alternative cost structures, ICERs varied proportionally with cost inputs while preserving the qualitative ranking of scenarios. Scenario analyses were deterministic and exploratory in nature and are intended to illustrate model responsiveness rather than to establish interaction effects.

### **European cost-environment transferability scenarios**

To evaluate cross-country transferability of cost results beyond the German reference setting, we constructed deterministic European cost-environment scenarios based on structured decomposition of incremental costs.

Total incremental cost in the base case was decomposed as:

$$\Delta Cost_{base}=\Delta Cost_{device}+\Delta Cost_{acute}$$

where:

- $\Delta Cost_{device}$ represents device acquisition and setup costs,
- $\Delta Cost_{acute}$ represents ICU and ward resource valuation.

Acute-care cost variation was modeled as:

$$\Delta Cost_{acute}^{(scenario)}=\Delta Cost_{acute}\times m_{acute}$$

with $m_{acute}=0.8$ and $1.2$, reflecting ±20% cross-country ICU cost variability. Device procurement variation was modeled as:

$$\Delta Cost_{device}^{(scenario)}=\Delta Cost_{device}\times m_{device}$$

with $m_{device}=0.85$ and $1.15$, reflecting ±15% European procurement variability.

Scenario-specific incremental costs were then calculated as:

$$\Delta Cost_{scenario}=\Delta Cost_{device}^{(scenario)}+\Delta Cost_{acute}^{(scenario)}$$

Incremental effectiveness was held constant:

$$\Delta QALY=0.95$$

and ICERs were derived as:

$$ICER_{scenario}=\frac{\Delta Cost_{scenario}}{\Delta QALY}$$

These scenarios were deterministic and designed to isolate cost-structure variability independent of clinical-effect uncertainty explored in probabilistic analyses.

## **Value-of-information (VOI)**

VOI analyses were conducted to quantify residual uncertainty in the base-case model and to prioritize parameters for future evidence generation. The EVPI represents the maximum value a decision-maker should be willing to pay to eliminate all model uncertainty, while the EVPPI isolates uncertainty attributable to specific parameter groups (Figure S3). At a WTP of €100 000 per QALY, the per-patient EVPI was €11 900, indicating moderate residual uncertainty surrounding ECPELLA’s economic value. Among parameter groups, the EVPPI was highest for device cost (€6 200) and survival benefit (€2 500), suggesting that further research should prioritize refining clinical effectiveness and device pricing evidence. Utilities, rehospitalization costs, and HF incidence contributed comparatively little to overall decision uncertainty (Figure S3).

**Figure S3. EVPPI by Parameter Group.** EVPPI values are shown per patient at a willingness-to-pay threshold of €100,000 per QALY. Device cost and survival effect were the dominant contributors to overall decision uncertainty.


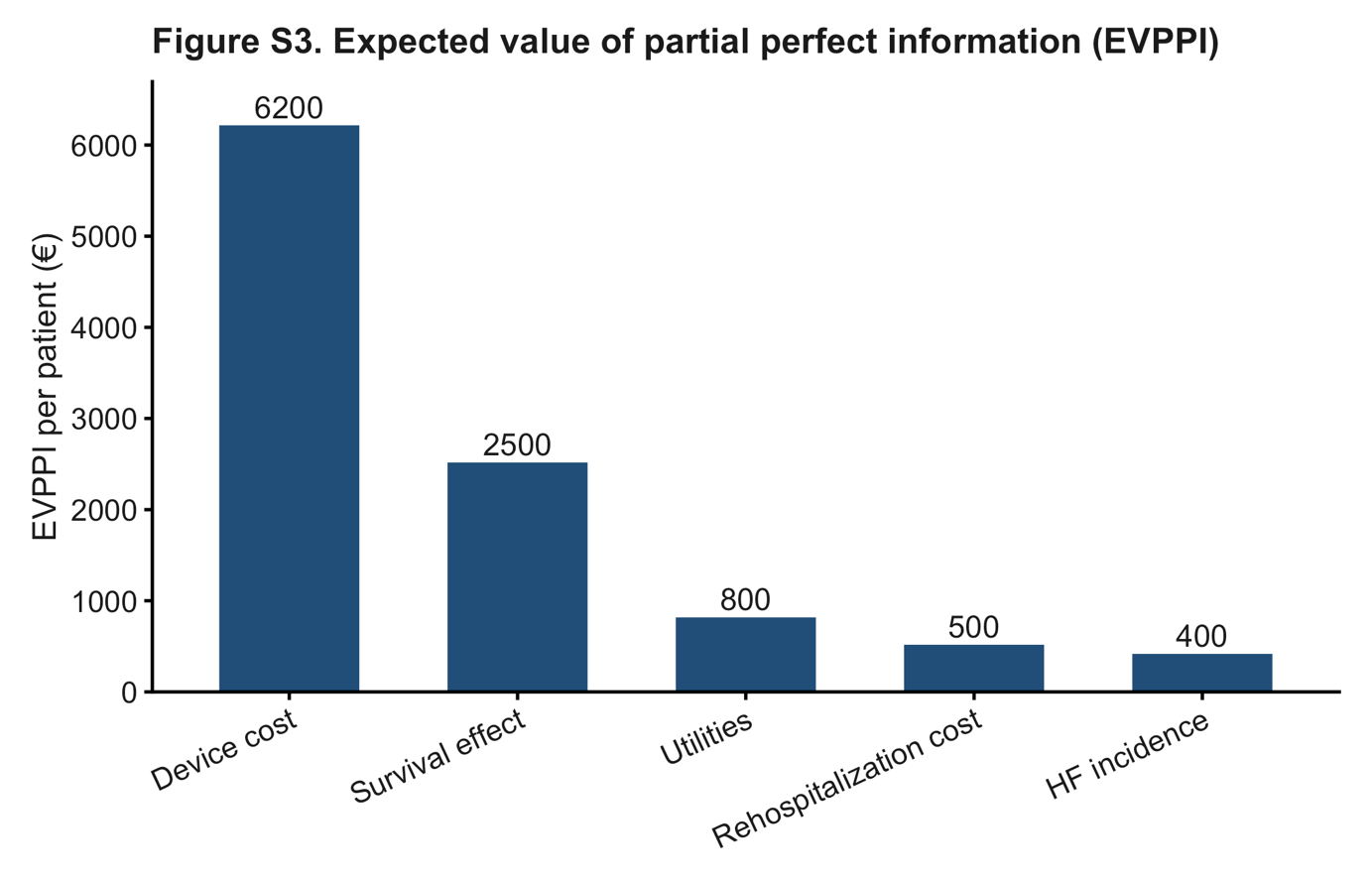


## **Budget impact and threshold analyses**

A five-year budget impact analysis (BIA) was conducted from the perspective of the German statutory health insurance system to estimate the projected financial consequences of ECPELLA adoption under progressive uptake scenarios. Assuming annual diffusion from 10% to 40% of eligible infarct-related CS patients, projected annual incremental expenditure reaches €64 million in year 5 (Table S6; Figure S4). Deterministic cost scenarios explored the influence of key acute-care cost drivers. A 20% reduction in device acquisition cost decreased projected year-5 expenditure to approximately €51 million. Reducing ICU length of stay by two days (base-case ICU per-diem €2,000) resulted in additional annual savings of approximately €4–6 million. Incremental cost-effectiveness threshold analyses were performed separately using a net monetary benefit framework to explore combinations of incremental cost and effectiveness associated with zero incremental net benefit. These analyses are reported in Figure S4 and do not alter the base-case clinical-effectiveness assumptions. Budget impact projections were calculated as uptake rate × eligible population × incremental per-patient cost derived from the 10-year base-case model.

**Figure S4. Budget impact of ECPELLA adoption.** The chart illustrates the projected annual incremental expenditure for the German statutory health insurance (GKV) under varying ECPELLA adoption rates among eligible infarct-related CS patients. Assuming an incremental per-patient cost of approximately €100 000, the total annual budget impact increases linearly from €8 million at 5 % uptake to €64 million at 40 %.

**
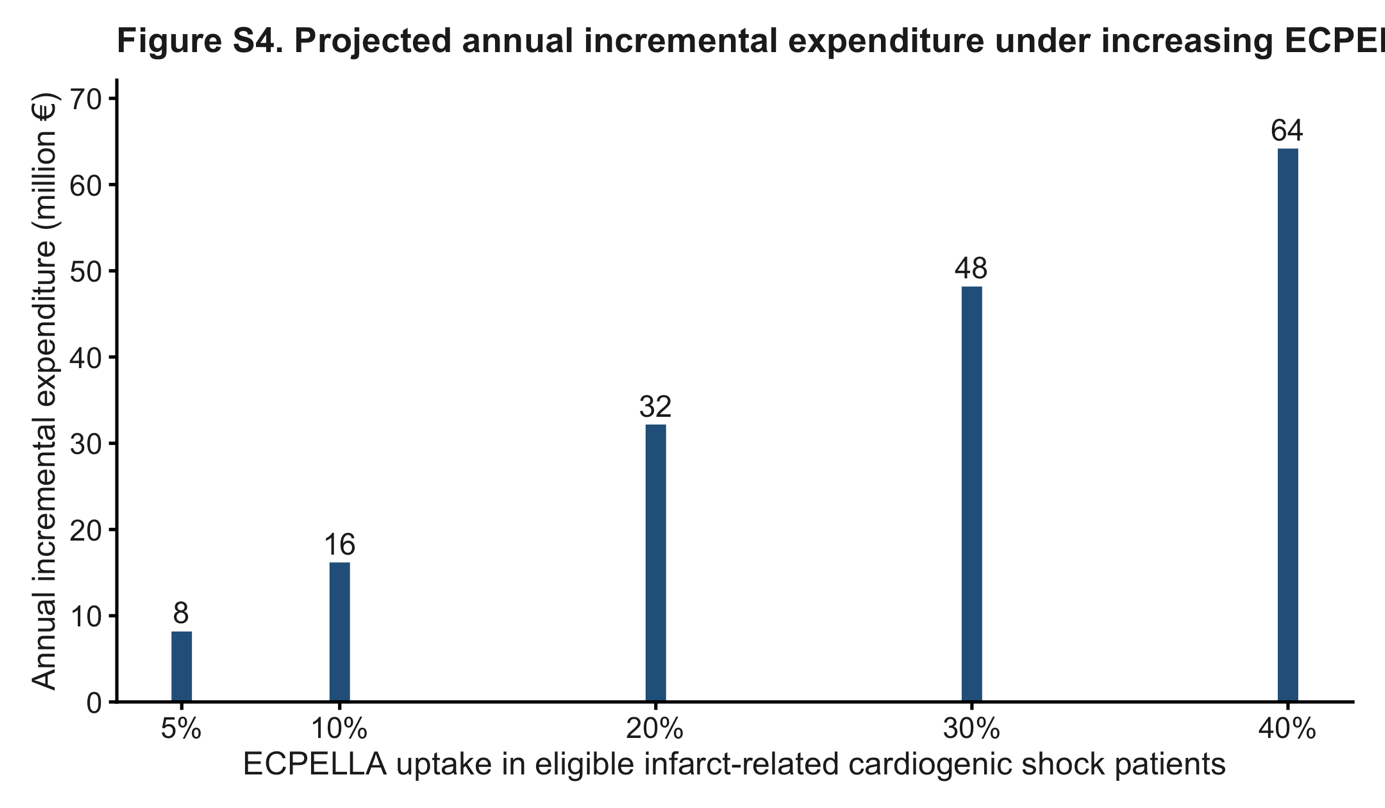
**

### **Table S6. Illustrative 5-year budget impact.**

| Year | Uptake (%) | Patients treated | Base case (€M) | Device −20% (€M) | ICU −2 days (€M) |
| --- | --- | --- | --- | --- | --- |
| 1 | 10 | 200 | 16.0 | 12.8 | 14.8 |
| 2 | 20 | 400 | 32.0 | 25.6 | 29.6 |
| 3 | 30 | 600 | 48.0 | 38.4 | 44.4 |
| 4 | 35 | 700 | 56.0 | 44.8 | 51.8 |
| 5 | 40 | 800 | 64.0 | 51.2 | 59.2 |

***Note****: Projected annual incremental expenditure associated with ECPELLA adoption from the perspective of the German statutory health insurance system. The base-case assumes an incremental per-patient cost of €100,000 derived from the 10-year model. Uptake rates represent hypothetical diffusion scenarios among eligible patients with infarct-related CS. The “Device −20%” scenario reflects a 20% reduction in acquisition cost of the microaxial flow pump. The “ICU −2 days” scenario assumes a reduction of two intensive care unit days per treated patient, based on a base-case ICU per-diem cost of €2,000. Estimates represent undiscounted annual incremental expenditure expressed in million euros (€M). No assumptions regarding clinical effectiveness were altered in these cost scenarios.*

**Data and code availability**

De-identified model input files, parameter distributions, and analytic code used for deterministic and probabilistic analyses are available from the corresponding author upon reasonable academic request for non-commercial, academic research purposes. Model development and analyses were conducted using R (version 4.3), Stata, and Microsoft Excel for data preparation, survival modelling, and probabilistic simulation. Reproducibility was ensured through version-controlled scripts and fixed random-number seeds for probabilistic analyses. External data used for calibration were obtained from published randomized trials and registries and are publicly accessible through their respective publications.

**CHEERS 2022 Reporting (Table S7)**

*Decision problem and setting*

This economic evaluation informs adoption decisions for ECPELLA (VA-ECMO + Impella unloading) versus GDMT in adult CS patients managed in tertiary ICUs and high-volume centers within the German statutory health insurance (GKV) system.

*Comparators*

GDMT was defined as contemporary intensive medical management for infarct-related CS without temporary mechanical circulatory support in the base-case analysis. GDMT comprised early revascularization, vasoactive and inotropic therapy, ventilatory support, renal replacement therapy where indicated, and intensive care monitoring. Structural sensitivity analyses explored alternative assumptions including rescue mechanical circulatory support; however, temporary MCS was not included in the base-case comparator.

*Time horizon and half-cycle correction.*

A 10-year base-case time horizon was applied to capture medium- to long-term survival and heart-failure burden following infarct-related cardiogenic shock. A lifetime (30-year) scenario was examined to assess long-term extrapolation effects. Half-cycle correction was applied to both costs and health outcomes.

*Perspective and included costs*

The analysis was conducted from a statutory health insurance payer perspective using a Western European acute-care costing framework. Detailed DRG-based and institutional micro-costing data from the German statutory health insurance system served as the reference environment for acute episode costs. Included direct medical costs comprised device acquisition and disposables, ICU and ward length of stay, revascularization procedures, renal replacement therapy, diagnostics, laboratory testing, and management of device-related complications and rehospitalizations. Indirect and out-of-pocket costs were not included.

*Currency, price date, and uprating*

All costs are reported in 2024 euros (€). Historical unit costs were adjusted to the 2024 price year using national health-sector inflation indices applicable to the reference costing environment. Where relevant, DRG-based reimbursement updates were applied to ensure internal consistency of acute-care cost inputs

*Measurement and valuation of effectiveness*

Short-term mortality (30-day and 180-day) and major adverse events were derived from contemporary randomized controlled trials and multinational cardiogenic shock registries. Baseline survival under guideline-directed medical therapy was calibrated to control-arm data and externally validated against registry mortality estimates. The relative treatment effect of ECPELLA was implemented using a proportional hazards framework based on the hazard ratio reported in the DanGer-Shock trial, applied to the calibrated baseline survival curve. Long-term survival beyond observed follow-up was extrapolated using parametric survival functions (Weibull and Gompertz distributions), selected based on statistical goodness-of-fit criteria and clinical plausibility. Excess mortality relative to age-matched population life tables was maintained during extrapolation.

*Heterogeneity*

Pre-specified subgroup analyses were conducted to explore structural and clinical heterogeneity. Subgroups were defined according to age (<50, 50–70, >70 years), underlying etiology (fulminant myocarditis, infarct-related cardiogenic shock, pulmonary embolism, postcardiotomy shock), timing of ECPELLA initiation (≤6 hours vs >24 hours from shock onset), and institutional case volume (high vs low).

Age-specific baseline mortality and life expectancy were adjusted using registry-derived estimates. Etiology- and timing-specific scenarios were modeled by varying survival benefit assumptions and acute-care resource use parameters. High-volume center scenarios incorporated reduced ICU length of stay and modified procedural cost inputs. Subgroup analyses were deterministic and exploratory and were not powered to establish interaction effects.

*Uncertainty*

Parameter uncertainty was addressed using probabilistic sensitivity analysis (PSA) with 10,000 Monte Carlo simulations. Beta distributions were applied to probabilities and utilities, gamma distributions to cost parameters, and log-normal distributions to relative treatment effects. Correlation between selected cost and length-of-stay parameters was incorporated where appropriate. Structural uncertainty was explored through alternative model specifications, including removal of the acute-phase tunnel state and testing alternative parametric survival extrapolation functions. Methodological uncertainty was assessed by varying the annual discount rate between 0% and 5% for both costs and health outcomes.

*Model availability*

De-identified model files and code are available upon reasonable academic request.

### **Table S7. CHEERS 2022 Checklist [62]**

| # | Item | Recommendation | Location in manuscript |
| --- | --- | --- | --- |
| 1 | Title | Identify study as economic evaluation (type, comparators) | Title page |
| 2 | Abstract | Structured summary: background, methods, results, conclusions | Abstract |
| 3 | Background/objectives | Context, decision problem, decision-maker | Introduction |
| 4 | Target population/subgroups | Describe population and subgroups | Methods → Population; S4 |
| 5 | Setting/location | Describe healthcare system and country | Methods → Perspective and setting |
| 6 | Study perspective | State and justify | Methods → Costs |
| 7 | Comparators | Describe and justify | Methods → Model overview |
| 8 | Time horizon | State and justify | Methods → Model overview |
| 9 | Discount rate | State and justify | Methods → Model; Costs |
| 10 | Choice of outcomes | Define outcomes and relevance | Methods → Economic outcomes |
| 11 | Measurement of effectiveness | Describe data sources | Methods → Clinical effectiveness |
| 12 | Valuation of preference-based outcomes | Describe utility measurement/valuation | Methods → Quality of life |
| 13 | Estimating resources and costs | Describe identification, measurement, valuation | Methods → Costs |
| 14 | Currency, price date | Report year and adjustments | Methods → Costs |
| 15 | Choice of model | Describe and justify | Methods → Model structure; Fig S1 |
| 16 | Assumptions | List structural and clinical assumptions | Methods; S2 |
| 17 | Analytic methods | Describe PSA/OWSA, correlations, calibration | Methods → Uncertainty analysis; VOI |
| 18 | Heterogeneity | Report subgroup methods and results | Results → Scenario analyses; S4 |
| 19 | Distributional effects | Equity/ethical considerations | Not applicable (not conducted) |
| 20 | Characterizing uncertainty | Describe parameter/structural/methodological uncertainty | Results → PSA; S5–S6 |
| 21 | Model validation | Report internal/external validation | Methods → Validation; S9 |
| 22 | Study parameters | Provide full parameter list | Tables S2–S3 |
| 23 | Summary of main results | Report Δcosts, ΔQALY, ICER, INMB | Results → Base case |
| 24 | Results: uncertainty | Report CEAC/intervals | Results → PSA; Fig S2; Table S5 |
| 25 | Results: heterogeneity | Report subgroup/scenario results | Table S4 |
| 26 | Discussion | Interpretation, limitations, generalizability | Discussion |
| 27 | Funding/conflicts | Report source and role | Declarations |
| 28 | Data/code | Report data/code access | Data/ Code Availability statement (Supplement) |

**References**

1. Thiele, H., et al., *Extracorporeal Life Support in Infarct-Related CS.* N Engl J Med, 2023. **389**(14): p. 1286–1297.

2. Ostadal, P., et al., *Extracorporeal Membrane Oxygenation in the Therapy of CS: Results of the ECMO-CS Randomized Clinical Trial.* Circulation, 2023. **147**(6): p. 454–464.

3. Lang, C.N., et al., *CS: incidence, survival and mechanical circulatory support usage 2007-2017-insights from a national registry.* Clin Res Cardiol, 2021. **110**(9): p. 1421–1430.

4. Morrow, D.A., et al., *American Heart Association CS Registry: Design and Implementation.* Circ Cardiovasc Qual Outcomes, 2024. **17**(7): p. e010637.

5. Lauridsen, M.D., et al., *Five-year risk of heart failure and death following myocardial infarction with CS: a nationwide cohort study.* Eur Heart J Acute Cardiovasc Care, 2021. **10**(1): p. 40–49.

6. Møller, J.E., et al., *Microaxial Flow Pump or Standard Care in Infarct-Related CS.* N Engl J Med, 2024. **390**(15): p. 1382–1393.

7. Patel, S.M., et al., *Simultaneous Venoarterial Extracorporeal Membrane Oxygenation and Percutaneous Left Ventricular Decompression Therapy with Impella Is Associated with Improved Outcomes in Refractory CS.* Asaio j, 2019. **65**(1): p. 21–28.

8. Bhatia, K., et al., *Meta-Analysis Comparing Venoarterial Extracorporeal Membrane Oxygenation With or Without Impella in Patients With CS.* Am J Cardiol, 2022. **181**: p. 94–101.

9. Schrage, B., et al., *Left Ventricular Unloading Is Associated With Lower Mortality in Patients With CS Treated With Venoarterial Extracorporeal Membrane Oxygenation.* Circulation, 2020. **142**(22): p. 2095–2106.

10. Bogerd, M., et al., *Impella and venoarterial extracorporeal membrane oxygenation in CS complicating acute myocardial infarction.* Eur J Heart Fail, 2023. **25**(11): p. 2021–2031.

11. Matsushita, K., et al., *Optimal Heart Failure Medical Therapy and Mortality in Survivors of CS: Insights From the FRENSHOCK Registry.* J Am Heart Assoc, 2024. **13**(5): p. e030975.

12. Hall, E.J., et al., *Survivorship After CS.* Circulation, 2025. **151**(3): p. 257–271.

13. Bandini, M., et al., *Midterm outcomes of patients with native heart recovery after Impella 5+ for CS.* Eur J Heart Fail, 2025. **27**(12): p. 3336–3341.

14. Ortuno, S., et al., *Long-Term Functional and Quality-of-Life Outcomes in Survivors of Refractory CS Treated With Venoarterial Extracorporeal Membrane Oxygenation.* Crit Care Explor, 2025. **7**(12): p. e1359.

15. Besnard, A., et al., *Association of VA-ECMO support and long-term quality of life after post-cardiotomy CS: experience from a prospective cohort.* Crit Care, 2026. **30**(1): p. 75.

16. Ludwig, K., J.G. von der Schulenburg, and W. Greiner, *Valuation of the EQ-5D-5L with composite time trade-off for the German population - an exploratory study.* Health Qual Life Outcomes, 2017. **15**(1): p. 39.

17. Ludwig, K., J.M. Graf von der Schulenburg, and W. Greiner, *German Value Set for the EQ-5D-5L.* Pharmacoeconomics, 2018. **36**(6): p. 663–674.

18. Delmas, C., et al., *Budget Impact Analysis of Impella CP(®) Utilization in the Management of CS in France: A Health Economic Analysis.* Adv Ther, 2022. **39**(3): p. 1293–1309.

19. Oude Lansink-Hartgring, A., et al., *Hospital Costs of Extracorporeal Membrane Oxygenation in Adults: A Systematic Review.* Pharmacoecon Open, 2021. **5**(4): p. 613–623.

20. Mishra, V., et al., *Cost of extracorporeal membrane oxygenation: evidence from the Rikshospitalet University Hospital, Oslo, Norway.* European Journal of Cardio-Thoracic Surgery, 2010. **37**(2): p. 339–342.

21. Jackson, C., et al., *A guide to value of information methods for prioritising research in health impact modelling.* Epidemiol Methods, 2021. **10**(1): p. 20210012.

22. Fenwick, E., B.J. O'Brien, and A. Briggs, *Cost-effectiveness acceptability curves--facts, fallacies and frequently asked questions.* Health Econ, 2004. **13**(5): p. 405–15.

23. Claxton, K., M. Sculpher, and M. Drummond, *A rational framework for decision making by the National Institute For Clinical Excellence (NICE).* The Lancet, 2002. **360**(9334): p. 711–715.

24. Fenwick, E., et al., *Using and interpreting cost-effectiveness acceptability curves: an example using data from a trial of management strategies for atrial fibrillation.* BMC Health Services Research, 2006. **6**(1): p. 52.

25. Robinson, A., et al., *Estimating a WTP-based value of a QALY: the 'chained' approach.* Soc Sci Med, 2013. **92**: p. 92–104.

26. Ryen, L. and M. Svensson, *The Willingness to Pay for a Quality Adjusted Life Year: A Review of the Empirical Literature.* Health Econ, 2015. **24**(10): p. 1289–1301.

27. Himmler, S., et al., *The value of health-Empirical issues when estimating the monetary value of a quality-adjusted life year based on well-being data.* Health Econ, 2021. **30**(8): p. 1849–1870.

28. Weinstein, M.C., et al., *Principles of Good Practice for Decision Analytic Modeling in Health-Care Evaluation: Report of the ISPOR Task Force on Good Research Practices—Modeling Studies.* Value in Health, 2003. **6**(1): p. 9–17.

29. Smare, C., et al., *Evaluating Partitioned Survival and Markov Decision-Analytic Modeling Approaches for Use in Cost-Effectiveness Analysis: Estimating and Comparing Survival Outcomes.* Pharmacoeconomics, 2020. **38**(1): p. 97–108.

30. Pavey TG, A.N., Taylor AH, et al. , *Economic modelling of cost-effectiveness*, in *The Clinical Effectiveness and Cost-Effectiveness of Exercise Referral Schemes: A Systematic Review and Economic Evaluation.*, A.N. Pavey TG, Taylor AH, et al. , Editor. 2011, NIHR Journals Library;.

31. Latimer, N.R., *Survival analysis for economic evaluations alongside clinical trials--extrapolation with patient-level data: inconsistencies, limitations, and a practical guide.* Med Decis Making, 2013. **33**(6): p. 743–54.

32. Eddy, D.M., et al., *Model transparency and validation: a report of the ISPOR-SMDM Modeling Good Research Practices Task Force-7.* Med Decis Making, 2012. **32**(5): p. 733–43.

33. Woods, B.S., et al., *Partitioned Survival and State Transition Models for Healthcare Decision Making in Oncology: Where Are We Now?* Value in Health, 2020. **23**(12): p. 1613–1621.

34. Titman, A.C., *Transition probability estimates for non-Markov multi-state models.* Biometrics, 2015. **71**(4): p. 1034–41.

35. Crowther, M.J. and P.C. Lambert, *A general framework for parametric survival analysis.* Stat Med, 2014. **33**(30): p. 5280–97.

36. Sinha Shashank, S., et al., *2025 Concise Clinical Guidance: An ACC Expert Consensus Statement on the Evaluation and Management of CS.* JACC, 2025. **85**(16): p. 1618–1641.

37. Heidenreich, P.A., et al., *2022 AHA/ACC/HFSA Guideline for the Management of Heart Failure: A Report of the American College of Cardiology/American Heart Association Joint Committee on Clinical Practice Guidelines.* Circulation, 2022. **145**(18): p. e895–e1032.

38. McDonagh, T.A., et al., *2021 ESC Guidelines for the diagnosis and treatment of acute and chronic heart failure: Developed by the Task Force for the diagnosis and treatment of acute and chronic heart failure of the European Society of Cardiology (ESC). With the special contribution of the Heart Failure Association (HFA) of the ESC.* Eur J Heart Fail, 2022. **24**(1): p. 4–131.

39. Werdan, K., et al., *[Short version of the 2nd edition of the German-Austrian S3 guidelines "CS complicating myocardial infarction-Diagnosis, monitoring and treatment"].* Anaesthesist, 2021. **70**(1): p. 42–70.

40. Werdan, K., et al., *Infarction-Related CS- Diagnosis, Monitoring and Therapy–A German-Austrian S3 Guideline.* Dtsch Arztebl Int, 2021. **118**(6): p. 88–95.

41. Jung, C., et al., *Management of CS: state-of-the-art.* Intensive Care Medicine, 2024. **50**(11): p. 1814–1829.

42. Moerer, O., et al., *A German national prevalence study on the cost of intensive care: an evaluation from 51 intensive care units.* Crit Care, 2007. **11**(3): p. R69.

43. Martin, J., et al., *[Cost of intensive care in a German hospital: cost-unit accounting based on the InEK matrix].* Anaesthesist, 2008. **57**(5): p. 505–12.

44. Kaier, K., et al., *Mechanical ventilation and the daily cost of ICU care.* BMC Health Serv Res, 2020. **20**(1): p. 267.

45. Srisawat, N., et al., *Cost of acute renal replacement therapy in the intensive care unit: results from The Beginning and Ending Supportive Therapy for the Kidney (BEST Kidney) study.* Crit Care, 2010. **14**(2): p. R46.

46. Rognoni, C., et al., *Impella Versus VA-ECMO for Patients with CS: Preliminary Cost-Effectiveness Analysis in the Italian Context.* Cardiol Ther, 2025. **14**(2): p. 183–198.

47. Thiele, H., et al., *Temporary mechanical circulatory support in infarct-related CS: an individual patient data meta-analysis of randomised trials with 6-month follow-up.* Lancet, 2024. **404**(10457): p. 1019–1028.

48. Shah, R.U., et al., *Post-Hospital Outcomes of Patients With Acute Myocardial Infarction With CS: Findings From the NCDR.* J Am Coll Cardiol, 2016. **67**(7): p. 739–47.

49. Thiele, H. and C. Hassager, *CS.* New England Journal of Medicine, 2026. **394**(1): p. 62–77.

50. Di Tanna, G.L., et al., *Health State Utilities of Patients with Heart Failure: A Systematic Literature Review.* Pharmacoeconomics, 2021. **39**(2): p. 211–229.

51. Schrage, B., et al., *From escalation to weaning strategies: how to integrate the ECMELLA concept.* Eur Heart J Suppl, 2023. **25**(Suppl I): p. I39–i43.

52. Gallen, R.A., et al., *Microcosting analysis of percutaneous coronary intervention with and without intracoronary imaging in an Irish tertiary referral centre.* Open Heart, 2025. **12**(1).

53. Fernando, S.M., et al., *Long-term mortality and costs following use of Impella® for mechanical circulatory support: a population-based cohort study.* Can J Anaesth, 2020. **67**(12): p. 1728–1737.

54. Ambrosy, A.P., et al., *The global health and economic burden of hospitalizations for heart failure: lessons learned from hospitalized heart failure registries.* J Am Coll Cardiol, 2014. **63**(12): p. 1123–1133.

55. Darbà, J., et al., *Economic burden of heart failure in Europe: A systematic review of costs and cost-effectiveness.* ESC Heart Fail, 2025. **12**(6): p. 4055–4068.

56. van den Berg, M., et al., *Hospital-related costs of sepsis around the world: A systematic review exploring the economic burden of sepsis.* Journal of Critical Care, 2022. **71**: p. 154096.

57. Foroutan, F., et al., *Global Comparison of Readmission Rates for Patients With Heart Failure.* J Am Coll Cardiol, 2023. **82**(5): p. 430–444.

58. Seferović, P.M., et al., *Insights into the European heart failure epidemiology.* Eur J Heart Fail, 2025. **27**(11): p. 1950–1960.

59. Schwalm, A., et al., *[IQWiG's methods for the cost-benefit assessment : Comparison with an international reference scenario].* Bundesgesundheitsblatt Gesundheitsforschung Gesundheitsschutz, 2010. **53**(6): p. 615–22.

60. Bojke, L., et al., *How to Appropriately Extrapolate Costs and Utilities in Cost-Effectiveness Analysis.* Pharmacoeconomics, 2017. **35**(8): p. 767–776.

61. Barman-Aksözen, J., et al., *Fair Funding Decisions: Consistency of the Time Horizons Used in the Calculation of Quality-Adjusted Life Years for Therapies for Very Rare Diseases by the National Institute for Health and Care Excellence in England.* Int J Environ Res Public Health, 2024. **21**(5).

62. Husereau, D., et al., *Consolidated Health Economic Evaluation Reporting Standards 2022 (CHEERS 2022) Statement: Updated Reporting Guidance for Health Economic Evaluations.* Value Health, 2022. **25**(1): p. 3–9.
